# Assessing evidence on the impacts of nature-based interventions for climate change mitigation: a systematic map of primary and secondary research from subtropical and tropical terrestrial regions

**DOI:** 10.1186/s13750-023-00312-3

**Published:** 2023-10-25

**Authors:** Samantha H. Cheng, Sebastien Costedoat, Amanda Sigouin, Gabriel F. Calistro, Catherine J. Chamberlain, Peter Lichtenthal, Morena Mills, A. Justin Nowakowski, Eleanor J. Sterling, Jen Tinsman, Meredith Wiggins, Pedro H. S. Brancalion, Steven W. J. Canty, Allison Fritts-Penniman, Arundhati Jagadish, Kelly Jones, Michael B. Mascia, Ana Porzecanski, Chris Zganjar, Carlos L. Muñoz Brenes

**Affiliations:** 1 World Wildlife Fund, 1250 24th St NW, Washington, DC 20037 USA; 2 The Betty and Gordon Moore Center for Science, Conservation International, 2011 Crystal Drive, Arlington, VA 22202 USA; 3 Center for Biodiversity and Conservation, American Museum of Natural History, New York, NY 10024 USA; 4 Department of Biology, Boston University, 5 Cummington Mall, Boston, MA 02215 USA; 5 The Nature Conservancy, Durham, NC 27701 USA; 6 Department of Ecology, Evolution, and Environmental Biology, Columbia University, 1200 Amsterdam Avenue, New York, NY 10027 USA; 7 Imperial College London, London, UK; 8 Smithsonian Environmental Research Center, 647 Contees Wharf Rd, Edgewater, MD 21037 USA; 9 Hawai’I Institute of Marine Biology, University of Hawai’i, Mānoa, HI, USA; 10 Independent Researcher, Washington, D.C., USA; 11 Department of Forest Sciences, ‘Luiz de Queiroz’ College of Agriculture, University of São Paulo, Piracicaba, Brazil; 12 Working Land and Seascapes, Smithsonian Institution, Washington, DC 20013 USA; 13 Saturday Academy, University of Portland, 5000 N Willamette Blvd, Portland, OR 97203 USA; 14 Human Dimensions of Natural Resources Department, Colorado State University, Fort Collins, CO 80523-1480 USA; 15 The Nature Conservancy, Arlington, VA 22203 USA

**Keywords:** Nature-based solutions, Natural climate solutions, Climate change mitigation, Carbon sequestration, Co-benefits, Impacts, Scale

## Abstract

**Background:**

Nature-based interventions (NbIs) for climate change mitigation include a diverse set of interventions aimed at conserving, restoring, and/or managing natural and modified ecosystems to improve their ability to store and sequester carbon and avoid greenhouse gas (GHG) emissions. Recent projections estimate that terrestrial NbIs can lead to more than one-third of the climate change mitigation necessary to meet the Paris Climate Agreement by 2030. Further, these interventions can provide co-benefits in the form of social and ecological outcomes. Despite growing recognition of the potential benefits, a clear characterization of the distribution and occurrence of evidence which supports linkages between different types of NbIs and outcomes for climate change mitigation, ecosystems, and people remains poorly understood.

**Methods:**

This systematic map assesses the evidence base on the links between NbIs and climate change mitigation, social, and ecological outcomes in tropical and subtropical terrestrial regions. We searched three bibliographic databases, 65 organization websites, and conducted backward citation chasing within 39 existing evidence syntheses to identify relevant articles. Additionally, we reached out to key informants for additional sources of evidence. We then used machine learning to rank returned results by relevance at the title and abstract stage and manually screened for inclusion using predefined criteria at the title, abstract, and full text stages. We extracted relevant meta-data from included articles using an a priori coding scheme. Lastly, we conducted a targeted, complementary search to identify relevant review and synthesis articles to provide broader context for the findings of the systematic map.

**Review findings:**

We included 948 articles in this systematic map. Most of the evidence base (56%) examined links between protection, natural resource management, and restoration interventions with changes to ‘proxy’ outcomes for climate change mitigation (changes to land condition, land cover, and/or land use). Other areas with high occurrence of articles included linkages between interventions within natural resource management and trees in croplands categories and changes to aboveground carbon storage and/or sequestration (17% of articles). A key knowledge gap was on measured changes in GHG emissions across all intervention types (6% of articles). Overall, articles in the evidence base did not often assess changes in co-benefits alongside direct or indirect changes for climate change mitigation (32%). In most cases, the evidence base contained studies which did not explicitly test for causal linkages using appropriate experimental or quasi-experimental designs.

**Conclusions:**

The evidence base for NbIs is significant and growing; however, key gaps in knowledge hamper the ability to inform ongoing and future investment and implementation at scale. More comprehensive evidence is needed to support causal inference between NbIs and direct outcomes for climate change mitigation to better determine additionality, permanence, leakage, and other unintended consequences. Similarly, priorities emerging from this map include the need for coordinated and harmonized efforts to collect diverse data types to better understand whether and how other outcomes (e.g. social, ecological) of NbIs can be achieved synergistically with mitigation objectives. Understanding potential benefits and trade-offs of NbIs is particularly urgent to inform rapidly expanding carbon markets for nature.

**Supplementary Information:**

The online version contains supplementary material available at 10.1186/s13750-023-00312-3.

## Background

**Fig. 1:**
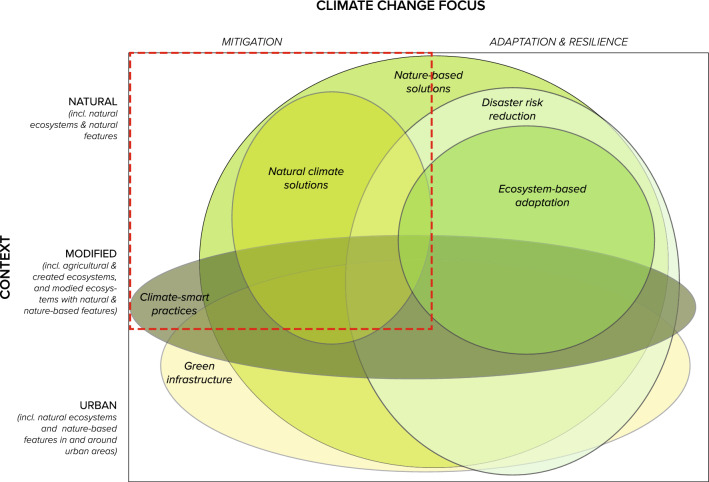
Conceptual alignment and overlap between different approaches, concepts, and frameworks for nature-based interventions for addressing climate change. This is a heuristic representation of how different concepts overlap, and is not indicative of breadth of scope. Natural habitats consist of areas where human activity has not essentially modified functions or composition (e.g. primary forest, streams, and wetlands). Modified habitats consist of areas where human activity has substantially modified ecological functions and/or species composition (e.g. agricultural lands, forestry lands) [14]. Urban habitats consist of natural ecosystems and nature-based features in and around urban areas [15]

Global efforts are accelerating to mitigate climate change impacts at scale. Natural and modified ecosystems play a crucial role in climate change mitigation as they remove carbon dioxide from the atmosphere—by sequestering (storing and capturing) carbon [1]. However, degradation and conversion of natural ecosystems (e.g. forest and grasslands) and production within the agricultural, forestry, and other land use (AFOLU) sectors contribute to about one-fifth of global greenhouse gas (GHG) net emissions [1]. Over the past few decades, interventions that work with and enhance nature to address societal challenges have gained traction as a set of cost-effective and scalable solutions to address climate change drivers and impacts [2–4]. These concepts, approaches, and frameworks (e.g. Nature-based Solutions (NbS), Natural Climate Solutions (NCS), Ecosystem-based Adaptation (EbA), Climate-smart practices for agriculture, forestry and husbandry, green Infrastructure, and Disaster Risk Reduction (DRR)) are often overlapping in objectives, focal environments, and intervention-types [3, 5] (see summary in [4, 6, 7]) (Fig. 1). Substantial attention has been paid to a subset of interventions that have demonstrated potential and/or specifically intend to reduce GHG emissions and increase carbon sequestration through protection, improved management, and restoration of ecosystems (e.g. [8, 9]). In this paper, we broadly refer to these interventions (e.g. Nature-Based Solutions [3] and Natural Climate Solutions [10]) as “Nature-Based Interventions” (NbIs) (Fig. 1). These interventions can also result in changes for social resilience, livelihoods, adaptive capacity, biodiversity maintenance, and other essential provisioning, regulating, and cultural ecosystem services [11–13].

Understanding and maximizing these other outcomes is fundamental for achieving the Sustainable Development Goals (SDGs) alongside the goals of the Paris Climate Agreement [16, 17] and the Kunming-Montreal Global Biodiversity Framework [18, 19]. However, reliable and relevant evidence is needed to understand what types of NbIs are well-suited to different social-ecological systems [20, 21] and how they can be designed to deliver and/or sustain outcomes for climate change, nature, and people. While there have been efforts to model and calculate the cost-effective climate change mitigation potential of different NbIs (e.g. [8, 10, 16, 22]) there has not yet been a cohesive effort to assess the state of evidence on the extent to which these outcomes are realized. Recent review efforts have examined discrete subsets of interventions (e.g. for forest conservation: [23–25]; for restoration in forests: [26–29]; for agroforestry: [30, 31]; for forest management: [32]; for conservation agriculture: [33]; and for monoculture plantations [34]).

In this paper, we first briefly discuss challenges for linking evidence to decisions about NbIs. Second, we describe a conceptual framework which informs the scope of this evidence map. Third, we characterize trends in the evidence base and discuss implications for research and practice in ongoing and future NbI efforts.

### Current challenges for understanding the effectiveness of nature-based interventions for climate change mitigation

Determining both potential and realized contributions to mitigation outcomes is challenged both by the reality of implementation, and the social-ecological contexts in which these interventions are embedded [35]. Interventions are rarely implemented in isolation. For example—integrated conservation and development approaches are often used to address social and ecological drivers of change [36–38]; and conservation agriculture interventions include practices that aim to reduce soil disturbance, maintain soil cover, and encourage crop rotation [33]. Moreover, interventions take place within broader socio-ecological systems with different interventions often implemented within the same landscape. These complexities make it challenging to disentangle attribution of effects at scale and the interactions between effects.

Other challenges for understanding effectiveness of NbIs include the range of methods and approaches for estimating emissions under different scenarios and measuring realized delivery of mitigation outcomes. Addressing these areas of inquiry requires reliable measurements of outcomes and robust study designs to reliably assess patterns of additionality, permanence, and leakage [39–41]. Thus, measures and study designs need to incorporate an appropriate counterfactual (i.e., answer the question “what would have credibly happened in the absence of the intervention?” [42]). Similar challenges arise for assessing NbIs’ social and ecological synergies and tradeoffs—despite considerable policy dialogue [43]. A systems perspective is required to monitor and evaluate both direct and indirect mechanisms from an intervention(s) to account for these dimensions themselves, as well as the relationships among them [44–46].

### Stakeholder engagement

As part of the process, the project convened and engaged a multi-sector and interdisciplinary stakeholder advisory group of researchers, practitioners, and policymakers who work in the sector. The stakeholder group provided input into the scope of this map specifically regarding elements of the synthesis questions, the framework of this synthesis, and suggestions for relevant literature and online sources of information (particularly grey literature). The stakeholder advisory group also provided guidance regarding interpretation of the insights from this map.

### Framework development

**Fig. 2:**
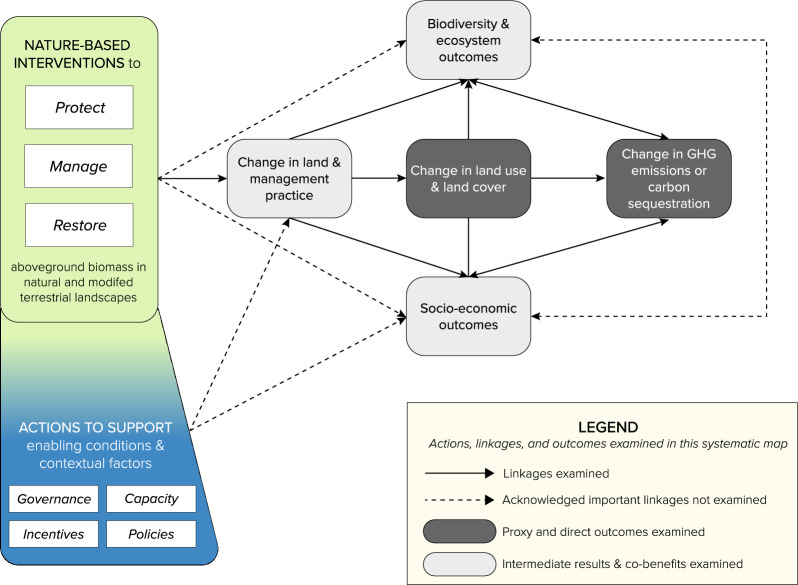
Working theory of change on the links between nature-based interventions (NbIs) for climate change mitigation (green box) and changes in greenhouse gas emissions and carbon sequestration outcomes via changes to aboveground biomass (measured as proxy outcomes—changes in land condition, land cover, and land use) in natural and modified terrestrial tropical habitats. These primary outcomes of interest are highlighted in dark gray. Complementary actions to support enabling conditions for NbIs are often carried out alongside or integrated with NbIs (blue box). Together, NbIs and complementary actions have other social and ecological outcomes (often described as co-benefits or co-impacts) (light grey boxes). However, we do not include articles that only focus on these other outcomes. Source: Cheng et al. 2022

The framework used in this study reflects a synthesis of existing conceptual models and causal theories on the links between NbIs, climate change mitigation outcomes, and other outcomes (e.g. [10, 24, 46]) within a unified, general theory of change (Fig. 2). In this study, we focus on a subset of NbIs with an explicit focus on mitigation objectives (which we refer to throughout as ‘NbIs for climate change mitigation’). This subset primarily includes NCS, with some exceptions (see Fig. 1 and “Methods” sections), some interventions which fall under the broader NbS framework, and some climate-smart practices. The rapid adoption and implementation of NbIs as a priority of climate change mitigation has raised concerns that there will be significant trade-offs for human well-being, nature, and equity without appropriate consideration of social and ecological contexts and conditions [47]. Increasingly, discussion on NbIs focuses on the influence of ‘complementary actions’ (i.e., interventions that aim to create or support enabling conditions), such as governance, rights, equity, resource tenure security [45, 47–54], and integration of people-centered actions (e.g. blue box, Fig. 2). For example, Other Effective Conservation Measures (OECMs) [55] seek to recognize and enhance the ability of Indigenous Peoples and Local Communities to exercise and retain rights to their traditional lands and resources, while delivering on targets for biodiversity, communities, and climate [56, 57]. In this study, we examine the extent to which existing evidence identifies when and how complementary actions are carried out alongside or integrated with NbI implementation.

In this systematic map, we considered both direct and ‘proxy’ outcomes for climate change mitigation (Table 1). We considered direct outcomes to be reported changes in GHG emissions (Mg CO_2_ and other GHG measured in Mg CO_2_ equivalents, or CO2e) and changes in carbon sequestration (in terms of change in storage and/or capture) in aboveground biomass. We considered “proxy” outcomes to be changes to land management practices (e.g. adoption) and land use and land cover change (LULCC) that either (a) covary with GHG emission and carbon storage; or (b) are intermediate outcomes along the pathway to climate change mitigation and are, therefore, indicative of future climate change mitigation potential [24]. Lastly, we include changes in land condition as a proxy for capacity for terrestrial ecosystems to sequester carbon. We focus on measures of change at the first point where carbon enters a terrestrial ecosystem (i.e. through aboveground biomass) and the last point where it exits (as emissions), thus capturing the primary ways in which changes to carbon storage and removal are commonly measured for tracking climate change mitigation at scale [58]. While other important biophysical interactions with climate exist (e.g. albedo, transpiration, biogenic volatile organic compounds), the interaction between these variables, land stewardship changes, and global climate change mitigation outcomes are beyond the scope of this analysis.

**Table 1:** Summary of intervention and outcome typology

Category	Definition
*Nature-based interventions for climate change mitigation*	
Protection	Establishing or expanding measures of protection for natural or semi-natural ecosystems for the purposes of conserving/regulating ecosystem services and natural landscapes/resources. Land or resource use is either fully restricted or significantly regulated. Specifically, actions intend to prevent conversion of forest or grasslands to croplands
Forest and other land use management (FOLU)	Actions directed at managing existing natural or semi-natural ecosystems OR created ecosystems for either the purposes of conserving/regulating ecosystem services and natural landscapes and/or providing sustained natural resources for use
Agricultural management	Actions directed at managing agricultural systems to mitigate climate change where possible—including climate or weather related risk (both extreme and slow-onset events), to improve food security in the short and long term. For NbIs, these actions should aim to be socially and culturally appropriate for the area where it is being practiced
Restoration	Actively re-establishing, enhancing, or establishing ecosystems to return them to natural or semi-natural states for the purposes of conserving/regulating ecosystem services and natural landscapes
*Climate change mitigation outcomes*	
Proxy outcomes	Changes in land condition (characteristics of ecosystems that affect its carbon storage potential); Changes in vegetation cover; Changes in land use
Direct outcomes	Change in emissions of greenhouse gasses (in metric tons of CO2eq); Changes in quantity of carbon stored in aboveground biomass and organic matter; Changes in the rate of carbon sequestration in aboveground biomass and organic matter
*Other outcomes*	
Adoption of practices/uptake	Related to the change in uptake of agricultural, land-use management, or forest management practices
Socioeconomic (co-benefit)	Related to changes in individual and/or collective well-being characterized by social and economic dimensions (economic well-being, health, safety and security, rights and empowerment, education and skills, social capital, culture) as well as agricultural productivity
Biological/ecological (co-benefit)	Related to changes in population, species, and /or community status, abundance, and/or structure; Outcomes related to changes in ecosystem function
Belowground carbon	Related to changes in belowground organic and inorganic carbon stocks and organic carbon sequestration rates

## Objective of the review

The primary objective of this systematic map is to identify, map, and describe the evidence base (including primary and secondary research) surrounding the impacts of NbIs on climate change mitigation outcomes and/or related LULCC impacts (or ‘proxy’ outcomes) in tropical and subtropical terrestrial ecosystems (including forests, grasslands, and mangroves). Given the wide range of potential audiences for this map, we aim to be broadly inclusive of articles and do not exclude articles based on study design, as we are not conducting critical appraisal of study reliability. In addition, this map does not seek to include or exclude articles based on the quality of intervention design or likelihood of success. Rather, this map aims to illustrate where there is significant research attention on different NbIs and characterize the articles therein.

A secondary objective of this study was to systematically map and describe the distribution of existing systematic maps, systematic reviews, and evidence gap maps that have focused on the links between NbIs and either climate change mitigation (direct and proxy) or co-benefit (socioeconomic, biological, and ecological) to provide context for the findings of our systematic map of primary articles. Thus, this mapping effort consists of two parts—the systematic map of primary articles focused on mitigation outcomes, and a complementary systematic map of reviews focused on mitigation outcomes, social, and/or ecological outcomes. This study addressed the following primary research question:

### What is the evidence base for links between NbIs and climate change mitigation outcomes in tropical and subtropical forests and grasslands ecoregions (including agricultural and native systems)?

This study also addressed the following set of secondary research questions:

- What is the extent and distribution of articles that examine climate change mitigation outcomes while also examining other outcomes (e.g. biodiversity/ecosystems, and/or human well-being outcomes)?
- What are the extent and distribution of articles that examine impacts on belowground climate change mitigation outcomes in addition to aboveground outcomes?
- What is the extent and distribution of articles that examine different mechanisms (e.g. political, policy, financial, institutional) through which interventions operate?
- What measures and methods are used to assess climate change mitigation outcomes?
- What is the extent and distribution of study designs being used to assess the impacts of NbIs on climate change mitigation?

## Methods

The scope and specific questions for this systematic map were commissioned by the Betty and Gordon Moore Center for Science at Conservation International (CI) to accelerate learning, inform the design, and support scaling of NbIs. The manuscript complies with CEE guidelines and standards and conforms to the ROSES reporting standards. As part of the process, a stakeholder advisory group was convened and the resulting insights informed the development of the protocol [59].

### Deviations from the protocol

The scope and methods for the systematic map of primary articles (objective 1) and the complementary map of reviews (objective 2) are described in the published protocol [59]. While our methods largely follow those outlined in the protocol, we did make a small number of refinements:

- We clarify that while the initial scope focused on NCS, the review team and the stakeholder advisory group realized that there was an opportunity to inform a broader audience and contribute towards evidence-informed practice across multiple sectors. As such, this map focuses on a suite of NbIs for climate change mitigation inclusive of NCS, along with other relevant practices from NbS and other climate-smart practices (Fig. 1, Table 1). We revised terminology from Natural Climate Solutions to Nature-Based Interventions for Climate Change Mitigation (NbIs) to refer to the scope of included interventions.
- We added Agris as an additional source for the search.
- For the systematic map, the final search string listed the following terms to filter for excluded populations (Boolean operator: NOT): United States, urban, city, cities, Japan, Argentina, South Africa, USA and peatland, which were not included in the protocol. While we excluded the terms “United States”, USA, and Japan—we did not exclude specific terms related to tropical and subtropical regions of these areas. Additionally, “community-based conservation area” was not in the protocol but added to the list of intervention terms.
- We excluded invasive species management as an NbI because, after additional review, invasive species management could not be reliably linked to climate change mitigation.
- We did not conduct full CEEDER scoring for all reviews. Instead, we used the following 3 criteria derived from CEEDER [71] for inclusion: reviews must (i) provide details on specific databases, search engines and/or organizational websites searched; (ii) list the search terms used; and (iii) include a separate list of articles included in the analysis.

### Search for articles

We undertook a comprehensive search strategy to capture an unbiased representation of existing literature (including both peer reviewed and grey literature) related to our research questions (Additional file 2). We searched bibliographic databases [Web of Science and Environment Complete (EBSCO)] in August 2021 and topical databases and organizational websites in October 2021; there was no search update. Given available resources for this systematic map we only performed searches in English. We followed the search process described in the protocol, with exceptions where noted above. We tested the comprehensiveness of the search string by testing it against a library of 30 relevant articles compiled by the review and stakeholder team and from backwards citation chasing. There was no search update. The final search strings are described in Additional file 2. Below is a set of search terms relevant to different components of the research question:

#### Population

(forest OR woodland OR meadow OR pasture OR agricultur* OR rangeland OR grassland OR mangrove OR tree OR cropland OR grazing OR land OR ecosystem OR landscape OR rice OR tropic*).

AND

#### Intervention

(restoration OR reforestation OR afforestation OR replanting OR rehabilitation OR enrichment OR "tree islands") OR ("rice production" OR "rice intensification" OR "rice cultivation" OR "community forest" OR "community forests" OR "community forestry" OR "shade grown" OR "climate-smart" OR "pasture management" OR "cover crop" OR "cover crops" OR "nutrient management" OR agroforestry OR agroforest OR silvopastor* OR silvopastur* OR silvo-pastor* OR silvo-pastur* OR agro-ecolog* OR agroecolog* OR "conservation agriculture" OR "tree planting" OR fencing OR exclosure OR ((partial OR selecti* OR gap OR retention) NEAR/3 (felling OR cutting OR harvest*)) OR "grazing management" OR "active management" OR "salvage logging" OR "reduced-impact logging" OR "alley cropping" OR "fire management" OR plantation OR "forest management" OR "manure management" OR ((crop OR cropland) NEAR/2 management) OR windbreaks OR thinning) OR ("protected area" OR "protected areas" OR ("Indigenous Peoples" OR "Indigenous communities" OR "Indigenous groups") OR "national park" OR "concession" OR "buffer zone" OR "sacred groves" OR "sacred forests" OR "sacred forest" OR "sacred grove" OR (protection NEAR/2 (forest OR landscape OR grassland))) OR ("land stewardship" OR "natural climate solutions" OR "natural climate solution" OR "ecosystem-based adaptation" OR "carbon forestry" OR "payments for ecosystem services" OR "payments for environmental services" OR "PES" OR "REDD" OR "REDD + " OR "Reduced Emissions from Deforestation and Degradation" OR "sloping land conversion" OR "cropland to forest").

AND

#### Outcome

("land use change" OR "land-use change" OR "land conversion" OR "forest conversion" OR "grassland conversion" OR deforestation OR "land cover" OR "forest cover" OR "vegetation cover" OR "habitat cover" OR "tree cover" OR (clearing NEAR/4 (forest OR land)) OR ((diversity OR composition OR recovery OR succession) NEAR/1 (tree OR forest)) OR ((biomass OR biomasses) NEAR/2 (tree OR shrub OR woody OR aboveground OR above-ground OR recovery OR living)) OR (degradation NEAR/2 (forest OR grassland)) OR ((climate OR carbon OR CO2 OR GHG OR "greenhouse gas") NEAR/3 mitigat*) OR ((carbon OR CO2) NEAR/2 (sequestration OR balance OR accounting OR storage OR emission OR sink OR stock OR fixation OR density)) OR (("greenhouse gas" OR GHG) NEAR/2 (emission OR avoid* OR reduc*)) OR aboveground OR above-ground).

AND

#### Outcome adjacent

(impact OR effect* OR evaluat* OR empiric* OR assess*).

NOT

(Canada OR "British Columbia" OR Europe OR Sweden OR Norway OR Finland OR Scandinavia* OR Mediterranean OR Chile OR "United Kingdom" OR Korea OR Pakistan OR Russia OR Denmark OR England OR Wales OR Ireland OR Scotland OR "integrated water resource management" OR European OR Spain OR Spanish OR Alabama OR Alaska OR Arizona OR Arkansas OR California OR Colorado OR Connecticut OR Delaware OR Florida OR Georgia OR Idaho OR Illinois OR Indiana OR Iowa OR Kansas OR Kentucky OR Louisiana OR Maine OR Maryland OR Massachusetts OR Michigan OR Minnesota OR Mississippi OR Missouri OR Montana OR Nebraska OR Nevada OR "New Hampshire" OR "New Jersey" OR "New Mexico" OR "New York" OR "North Carolina" OR "North Dakota" OR Ohio OR Oklahoma OR Oregon OR Pennsylvania OR "Rhode Island" OR "South Carolina" OR "South Dakota" OR Tennessee OR Texas OR Utah OR Vermont OR Virginia OR Washington OR "West Virginia" OR Wisconsin OR Wyoming OR "north america" OR "north american" OR Albania OR Andorra OR Armenia OR Austria OR Azerbaijan OR Belarus OR Belgium OR Bosnia and Herzegovina OR Bulgaria OR Croatia OR Cyprus OR Czechia OR Estonia OR France OR Germany OR Greece OR Hungary OR Iceland OR Italy OR Kazakhstan OR Kosovo OR Latvia OR Liechtenstein OR Lithuania OR Luxembourg OR Malta OR Moldova OR Monaco OR Montenegro OR Netherlands OR Poland OR Portugal OR Romania OR Russia OR San Marino OR Serbia OR Slovakia OR Slovenia OR Switzerland OR Turkey OR Ukraine OR "Vatican City" OR Alberta OR "British Columbia" OR Manitoba OR "New Brunswick" OR Newfoundland OR "Northwest Territories" OR "Nova Scotia" OR Nunavut OR Ontario OR "Prince Edward Island" OR Quebec OR Saskatchewan OR Yukon OR Labrador).

NOT

TI = ("modelling" OR "modeling").

### Screening process

We compiled the articles retrieved from the methods described above and assessed articles for inclusion using colandr (http://www.colandrapp.com) [61]. First, we screened all records at the title and abstract level; records that met the criteria (or for which it was unclear) were included for full-text screening. We recorded all screening decisions and reasons at title/abstract and full text level. We added records flagged as reviews or syntheses to the map of reviews screening process (see Additional file 1). We performed double screening to test screening consistency at title/abstract level and full text; consistency checking was conducted using a two-step, double-blind method. For the main map, two reviewers screened 11% of records at title and abstract (1500 items), and 10% of records screened at full text (218 items); for the map of reviews, two reviewers screened approximately 15% of records at both stages (420 and 115 items, respectively). All screening inconsistencies (~ 15% of double-screened items) were discussed and reconciled across the team (resulting in 100% consistency). Reviewers did not screen nor code articles that they were authors on.

### Eligibility criteria

We applied the following criteria at the title and abstract level and again at the full text level.

#### Population

- We focused on ecoregions within the following tropical and subtropical terrestrial biomes: Tropical and Subtropical Coniferous Forests; Tropical and Subtropical Dry Broadleaf Forests; Tropical and Subtropical Grasslands, Savannas and Shrublands; Tropical and Subtropical Moist Broadleaf Forests; Mangroves [60].

#### Primary intervention

- We included interventions across three broad themes—protection, management, and restoration. We distinguish between forest and other land use management and agricultural management. See Additional file 4 for full typology of included interventions.

#### Study type(s)

We included primary research articles in English that met the following criteria:

- Non-experimental, quasi-experimental, and experimental study designs that use quantitative data, qualitative data, or a combination of data types. We categorized study designs by assessing components of study design (treatments, controls, comparisons) (Additional file 2).
- Systematic reviews and syntheses (e.g. systematic maps, evidence gap maps, meta-analyses) that describe the methods used for search, data collection, and synthesis and provide a list of included articles.

We excluded the following study types:

- Theoretical or modeling studies, editorials, and commentaries.
- Literature reviews that do not describe methods used for search, data collection, and synthesis or do not provide a list of included articles.

#### Outcomes

- We included articles that assess changes in GHG emissions, amount of carbon stored (carbon storage), and/or rates of carbon sequestration within aboveground biomass as an indicator of climate change mitigation. We also include articles that assess changes to land cover, land use type, and/or land condition as proxy indicators as they represent intermediate outcomes on the pathway towards mitigation. See Additional file 5 for a full typology of included outcomes.
- We included articles that assess changes in behavioral, socio-economic, biological, and/or ecological outcomes in addition to changes in mitigation outcomes described above.

### Study validity assessment

Given the large size of this systematic map, we did not undertake a quality assessment for individual articles in terms of reliability and relevance based on study design. We do code study design and comparators from each included article to provide a heuristic assessment of study reliability.

### Coding strategy

We used a standardized data coding form to extract relevant information for each article for the main map (Additional file 6). We extracted meta-data in Knack. Two reviewers coded 5% of included articles with side-by-side double coding. Reviewers met to discuss consistency checks for all double-coded articles resulting in 100% consistency after discussion. Additionally, random spot checks of 30 articles were conducted by a second reviewer to ensure consistency of single reviewer coded articles (any discrepancies were discussed and adjusted, resulting in 100% consistency in this set). Any other inconsistencies in coding were discussed among the coding team and adjusted as needed. If any information was missing or unclear, we left it uncoded as the volume of items was too large to contact authors to obtain this information. The extracted data included:

- Unique article ID and assessor information.
- Bibliographic information.
- Intervention type and details.
- Any complementary interventions implemented alongside or in concert with the intervention (and details).
- Study location, scale, design, and comparator details.
- Outcome type and details.
- Details on categories of co-benefits evaluated (socioeconomic and/or biological/ecological outcomes, belowground outcomes, changes to practice/adoption/uptake).

### Data mapping method

We mapped the distribution of the evidence base as heatmaps with individual cells that depict the number of articles that examined linkages between interventions and outcomes. Given articles can examine more than one intervention and/or outcome, a single article may appear in more than one cell of the heatmap. In cases where two or more articles examined the same study, we would include the most comprehensive and recent article. We used the heatmaps to identify evidence clusters and gaps for both the systematic map and the map of reviews.

## Review findings

### Review descriptive statistics

**Fig. 3:**
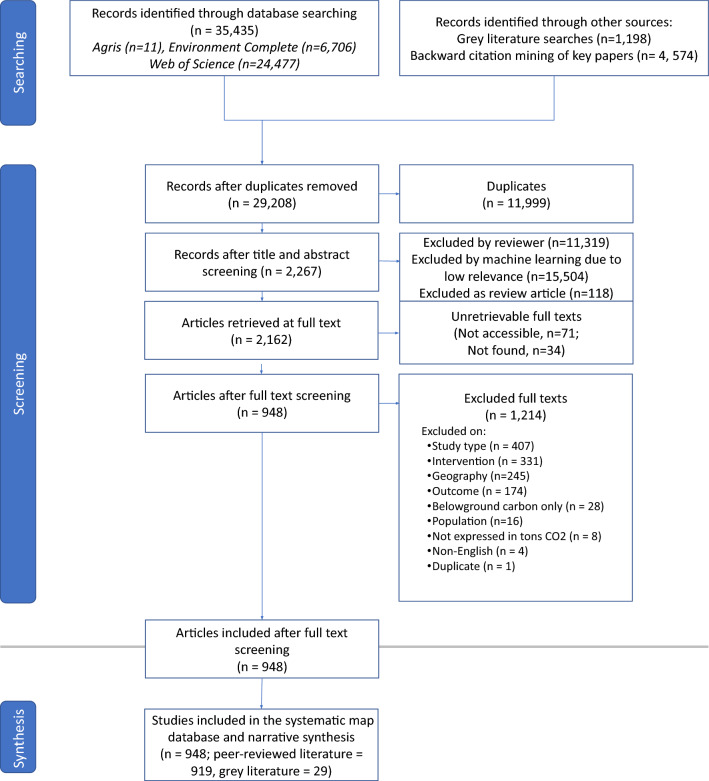
ROSES flow diagram illustrating articles recovered in initial search and inclusion and exclusion statistics through screening and full text assessment. ROSES flow diagram for map of reviews is in Additional File 9: Fig. S1

Our search identified 35,435 peer reviewed and 1198 grey literature articles that were potentially relevant for the systematic map; we removed 11,999 duplicate citations in Endnote matching based on title. We used the machine learning algorithm employed in colandr to sort articles by relevance and stopped screening titles and abstracts when our rate of included articles dropped below 5% per 300 citations screened based on a heuristic estimate of likely return of relevant articles. We screened 13,704 articles for inclusion based on simultaneous reading of title and abstract, of which, 2,267 were included for full text screening. Full texts were retrieved using Columbia’s institutional subscriptions and through Google. We were unable to access 71 articles and could not find 34 articles (~ 5% of full texts. Ultimately, 948 articles (919 peer-reviewed and 29 grey-literature reports) were included in the final systematic map (Fig. 3) (Additional file 7). At the full text screening stage, most articles were excluded due to study type (~ 30% of excluded articles) followed by intervention type (25%) (see Additional file 8). For the map of reviews, we identified 5251 articles, 769 of which were examined at full text, ultimately including 319 review and synthesis articles that met our criteria. We separated these articles into two categories: (1) articles that reviewed *only* climate change mitigation outcomes (proxy outcomes and/or storage, sequestration, and emission outcomes) (97 articles); and (2) articles that reviewed socioeconomic, biological/ecological, and human behavior change outcomes (alone or in addition to climate change mitigation outcomes) (222 articles) (Additional file 9).

A bibliography of included articles in the map of primary research and a bibliography for included articles in the map of reviews are in Additional file 7. A bibliography of articles excluded at full text from the map of primary research and the map of reviews, including reasons for exclusion, is listed in Additional file 8. Coded data for all included articles in the map of primary research are included in Additional file 6 and in Additional file 10 for the map of reviews. RepOrting standards for Systematic Evidence Syntheses (ROSES) reporting forms (for both the map of primary research and map of reviews) are included in Additional file 11.

### Characteristics of the evidence base

**Fig. 4:**
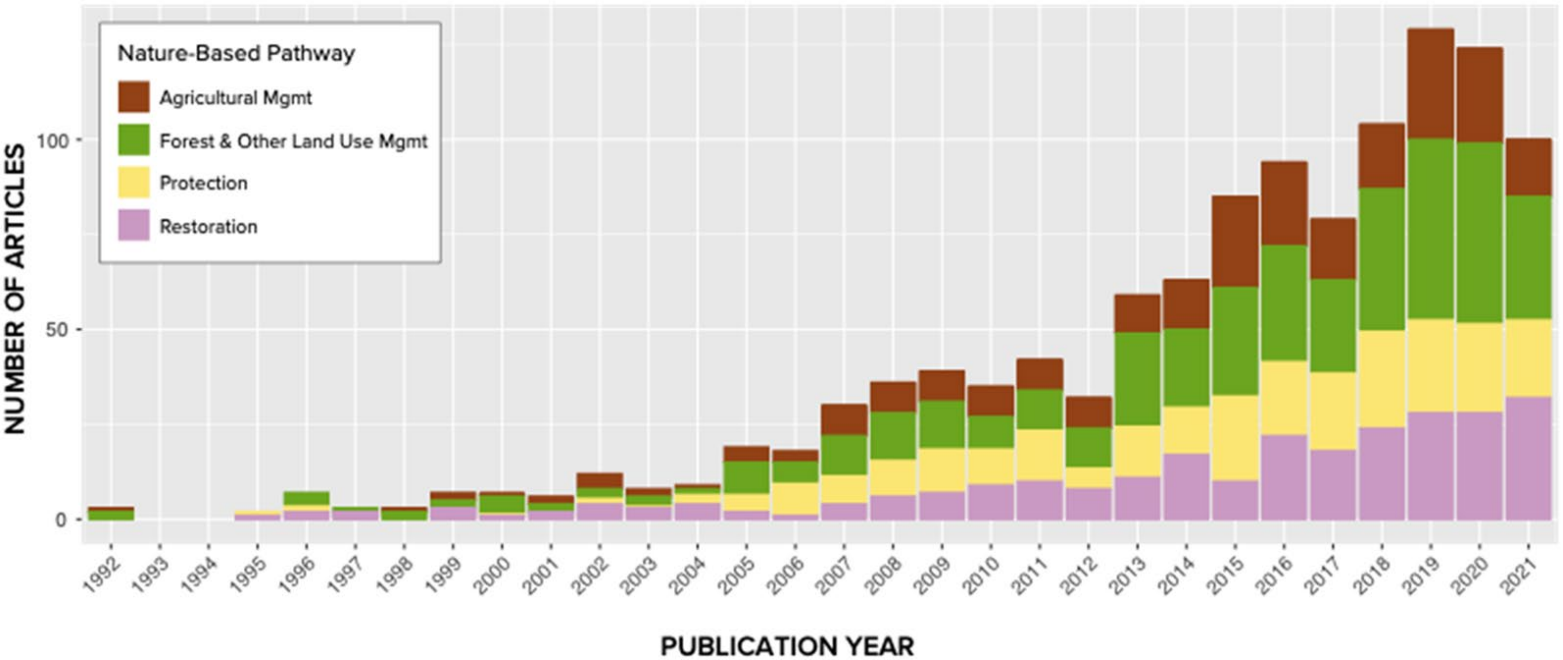
Growth in evidence base from the systematic map disaggregated by intervention pathway

Since the early 1990s, the number of primary articles published annually has increased, with reviews beginning to emerge around the 2010s (Additional file 9: Fig. S2). Attention on NbIs has grown over the last 30 years; with an increase in articles examining the Forest and Other Land Use and Management pathway beginning in 2013 while articles on restoration have grown more slowly since the late 2000s (Fig. 4). Most first authors were affiliated with an academic institution (83% in the systematic map, 77% in the map of reviews) (Additional file 9: Fig. S3).

**Fig. 5:**
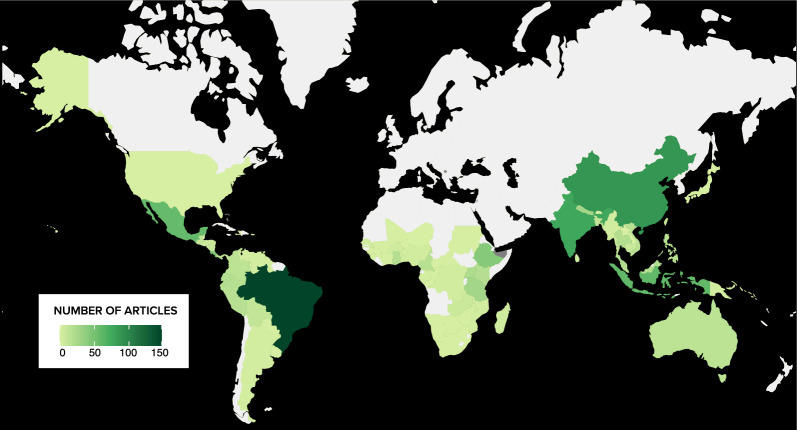
Geographic distribution of articles across countries under study in the systematic map. Entire countries are indicated in this map—however, articles are not evenly distributed across all parts of a country (e.g. may have specific local or sub-national focus)

Most articles evaluated interventions at the sub-national and local scales (Additional file 9: Fig. S5) with many focusing on interventions in Latin America (n = 381, 40%), followed by Asia (n = 370, 39%), and Africa (n = 217, 23%); with far fewer articles focused on countries in Oceania (n = 20, 2%) (Fig. 5). We found certain countries were the focus of a relatively higher number of articles; for instance, three countries alone—Brazil (n = 147), China (n = 88), and India (n = 76)—were the focus of 47% of total primary articles. Other countries in South America (outside of Brazil) and Africa are relatively less studied, reflecting overall publication biases found in other studies in conservation topics (e.g. 62). The low number of articles documenting interventions in places such as United States of America (n = 1) and Australia (n = 15) is likely explained by our study focusing only on tropical and subtropical regions, which cover a small percentage of land in those countries (e.g. Hawai’i). Notably, China had the second highest number of articles even though tropical and subtropical terrestrial lands only comprise a small percentage of its territory (~ 26%, [63]).

**Fig. 6:**
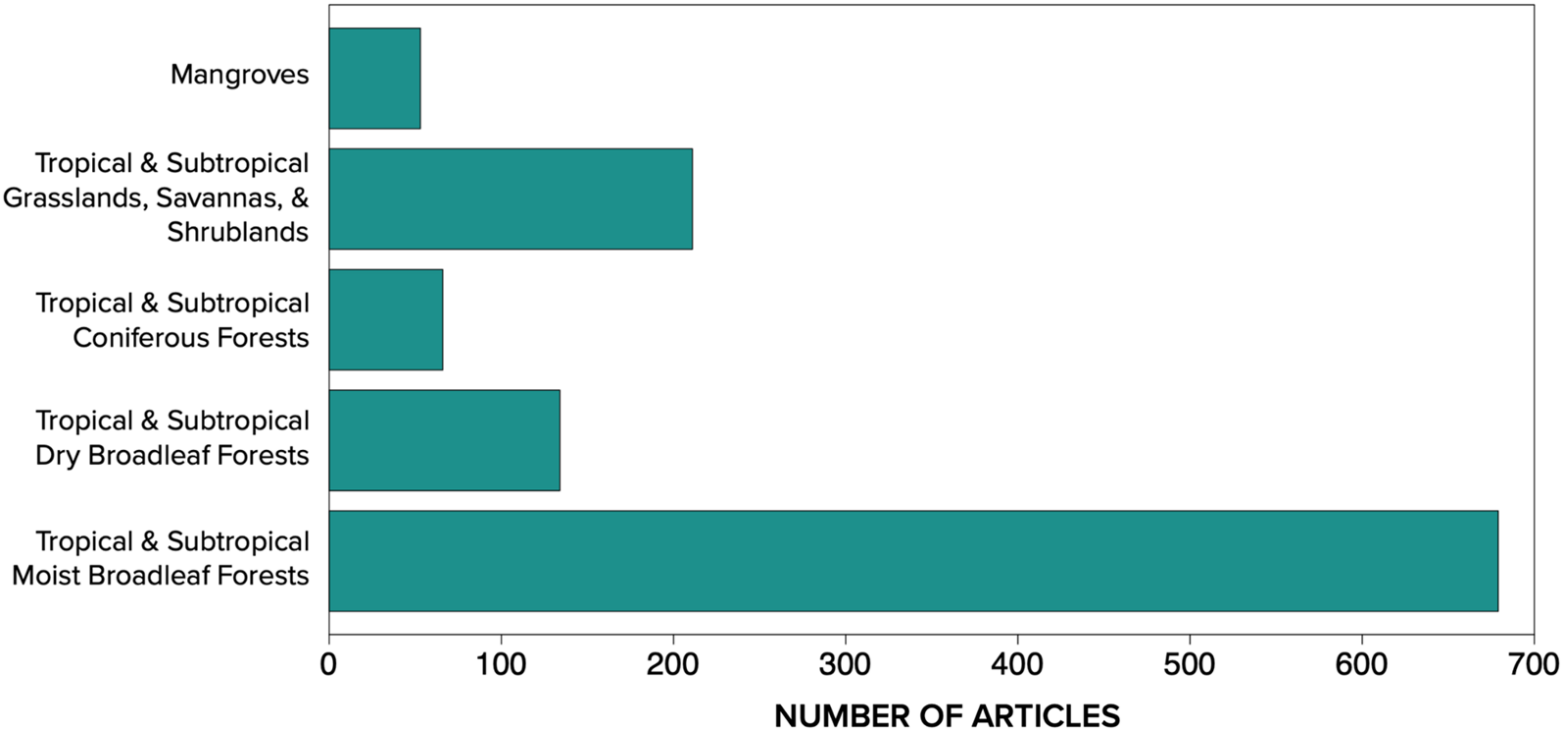
Distribution of peer-reviewed published articles and grey literature across biomes where studies took place. Articles can appear in more than one biome type

Most articles evaluated interventions taking place within forests, with the majority focused on Tropical and Subtropical Moist Broadleaf Forests (n = 679) and comparatively far fewer articles examining interventions within grasslands, savannas, and shrublands (n = 211) (Fig. 6). NbIs in mangroves were the least studied biome type (n = 53), which may point to an important evidence gap as mangroves are among the ecosystems containing the highest reserves of irrecoverable carbon [64].

### Trends across nature-based interventions for climate change mitigation

#### Nature-based interventions

**Fig. 7:**
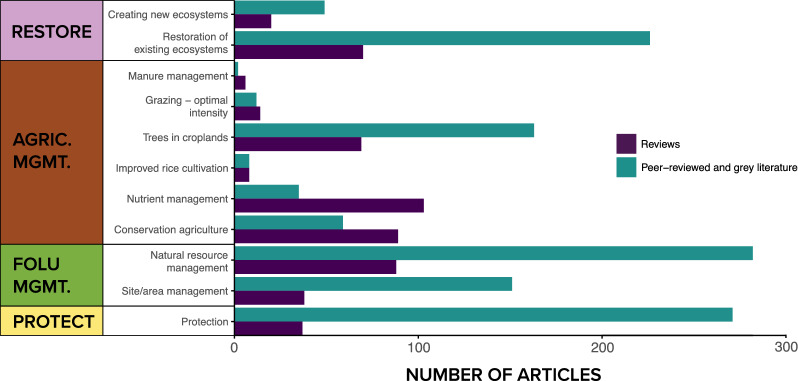
Distribution and frequency of interventions examined across intervention categories. Articles and reviews can appear in more than one intervention type. *PROT* protection, *FOLU* *MGMT* forest and other land use management, *AGRIC* *MGMT* agricultural management, *RESTORE* restoration

The greatest number of articles focused on natural resource management activities (n = 282, 30% of included articles), followed by protection (n = 271), and restoration of existing ecosystems (n = 226) (Fig. 7). Within the agricultural management pathway, most articles assessed ‘trees in croplands’ (n = 163, 17%) interventions, with a significant focus on agroforestry and silvopastoral activities. Within restoration, the majority of articles were focused on restoring existing ecosystems (n = 226, 24%) versus creating new ecosystems, e.g. afforestation (n = 49, 5%). The patterns seen in primary articles were largely reflected in the review articles, however, there were significantly more review articles focused on nutrient management (n = 103) and conservation agriculture (n = 89) (that include tropical and subtropical terrestrial biomes) than were recovered in the systematic map of primary articles, indicating that attention to these intervention types within the geographic scope of this systematic map is lower than the evidence base that exists globally (Fig. 7).

Research attention on categories of NbIs is not evenly distributed across biomes nor regions of the world. For example, approximately half of mangrove-focused articles evaluated restoration interventions, while most articles focused on forests and grasslands evaluated protection and management interventions (Additional file 9: Table S1). In Africa, there is a relatively higher concentration of agricultural management articles and a relatively lower quantity of restoration articles (Additional file 9: Table S2). Most articles examined one intervention type (n = 691, 73%). Of the articles that did examine more than one NbI (n = 257, 27%), the articles focused on comparing outcomes between different intervention types, rather than the simultaneous evaluation of multiple interventions.

#### Complementary actions

Overall, ~ 18% of the evidence base (n = 168 articles) described complementary actions implemented alongside NBIs to address enabling conditions. Most articles focused on actions related to strengthening rights to resources (n = 68), policies and regulations (n = 61), strengthening participation and empowerment (n = 56), and livelihood/economic resilience (n = 46) (Additional file 9: Fig. S7). This trend is also reflected in the distribution of review articles (e.g. strengthening rights to resources, n = 15 reviews). In comparison, while the evidence base did not contain many articles examining the influence of informal or formal education within the context of NbIs, several reviews exist on these complementary actions, albeit not only focusing on mitigation outcomes.

**Fig. 8:**
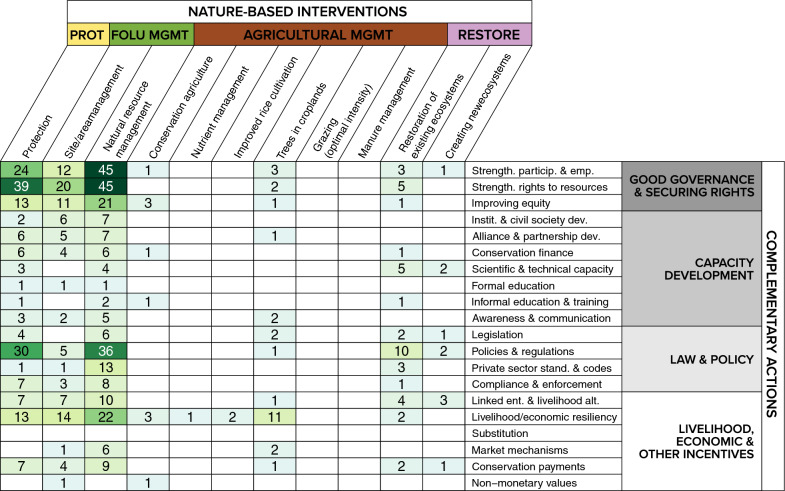
Heatmap of nature-based interventions and complementary actions examined within peer-reviewed and grey literature articles. *PROT* Protection, *FOLU* *MGMT* forest and other land use management, *AGRICULTURAL* *MGMT* agricultural management, *RESTORE* Restoration

Within the evidence base, articles most frequently evaluated natural resource management interventions alongside complementary actions related to (i) strengthening participation and empowerment, and (ii) strengthening rights to resources (Fig. 8). Another relatively well-studied area is between protection and strengthening rights to resources. Notably, complementary actions were not often examined within the context of agricultural management interventions. Similarly, review articles also rarely explicitly examined complementary actions (Additional file 9: Fig. S7). Of those that did, they most often looked at complementary actions related to strengthening rights to resources (n = 8 reviews), improving equity in conservation processes and delivery of benefits (n = 6 reviews), and conservation payments (n = 5 reviews).

#### Study design

**Fig. 9:**
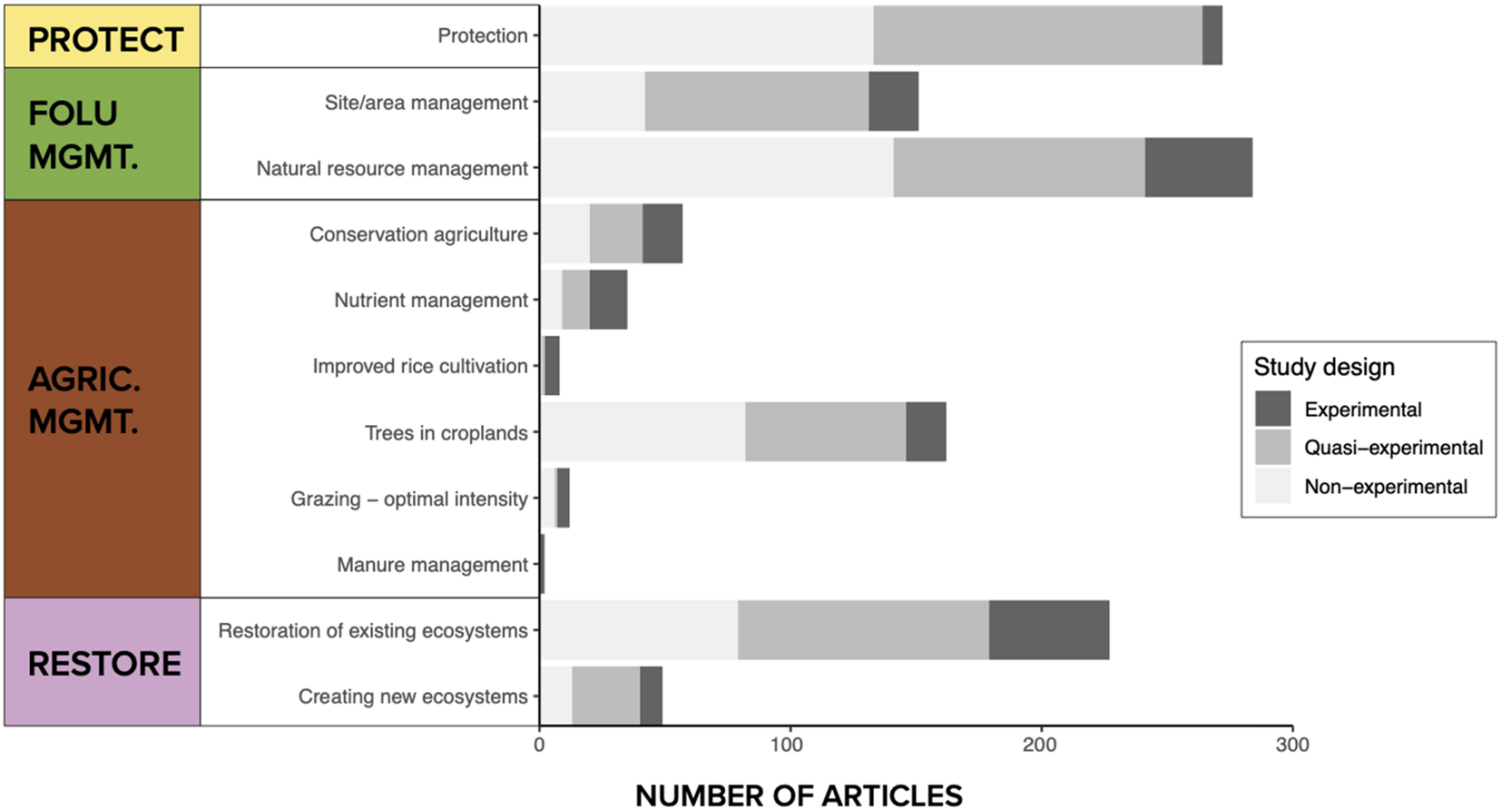
Study designs employed in the systematic map across interventions studied. *PROT* protection, *FOLU* *MGMT* forest and other land use management, *AGRIC* *MGMT* agricultural management, *RESTORE* restoration

Nearly 57% of included articles (n = 538) compared changes to a control (i.e. quasi-experimental) and/or employed an experimental design, suggesting that there may be potential to infer causal relationships between NbIs and climate change mitigation outcomes from a sizable subset of the current evidence base. Notably, while there were few articles in the evidence base on manure management and grazing management, most were experimental and quasi-experimental (Fig. 9). However, it should be noted that experimental articles on interventions in the agricultural, forests, and other land use management pathways were primarily experiments in closed systems which may have lower external validity to inform implementation in real-life situations. Articles most frequently compared measured outcomes across presence/absence of an intervention (n = 392, 41%) (Additional file 9: Fig. S4, Table S4) across all study designs. However, as this map did not aim to assess the quality of study designs and strength of evidence (e.g. level of replication, and nature and reliability of controls), but rather the stated comparisons and methods used, this finding should be interpreted carefully.

#### Cost reporting

Few articles reported costs related to implementing NBIs alongside outcome measurements (n = 54 out of 958 articles, 6%). Cost reporting has somewhat increased since 2015, however, this pattern is not consistent relative to overall articles for each consecutive year (Additional file 9: Fig. S8).

Types of costs were assessed for 47 articles across categories of possible costs incurred including implementation cost and maintenance cost, among others (Box 1). Implementation cost (n = 40 articles) and cost-effectiveness of interventions (n = 34 articles) were most reported. For example, cost-effectiveness was often reported as comparisons of costs between different types of interventions. Most articles reported two to three different types of costs (n = 31 articles) and costs were evenly reported across all NbIs. Notably, 17 articles reported costs associated with Payments for Ecosystem Services (PES) schemes, most often in terms of payments made to participants.

**Box 1:** Types of cost

**Implementation cost:** Costs incurred from the implementation of the intervention, i.e. labor, transportation, materials, infrastructure, training, and payment to participants (if applicable)
**Cost-effectiveness:** Expressed as cost per standardized spatial unit and/or standardized unit of sequestered carbon; or cost comparisons between different types of interventions
**Opportunity cost:** Benefits forgone as a result of engaging in an NBI, commonly expressed as the value of opportunities or net benefits forgone
**Sustainability/permanence cost:** Post-implementation monthly or annual costs for continuing the intervention
**Monitor/maintenance cost:** Costs for data collection, analysis, validation, and management to monitor the progress and impacts of the intervention over a landscape

Interventions relating to agroforestry presented several common types of cost data. Agroforestry studies discussed costs associated with the implementation of the intervention in the form of fertilizer, weeding, and fencing (n = 9). Additionally, in these articles cost-effectiveness (n = 7) was presented as frequently as implementation cost (n = 7). Cost-effectiveness appeared as an overarching goal behind interventions with the aims of efficiency and creating equivalent or greater returns for NbIs than for non-sustainable agricultural methods. Cost comparisons were most often reported from agricultural studies, either comparing different management methods (e.g. organic vs. traditional agriculture) or different types of crops. In these comparisons, costs were typically presented as a cost per unit (e.g. USD per hectare or by USD per ton of sequestered carbon). Articles examining restoration (n = 13) frequently compared cost-effectiveness of different approaches for sequestering carbon. Restoration costs included expenses to implement the restoration intervention (e.g. cost of seedlings and/or necessary labor), as well as costs for monitoring of the results.

### Trends in climate change mitigation outcomes and other impacts

We examined four core climate change mitigation outcomes: land condition, land use/land cover, carbon storage and/or sequestration, and greenhouse gas emissions. We also assessed whether articles reported on other outcomes within the broader social-ecological system including human behavior change, co-benefits (socioeconomic and ecological changes), and/or changes to belowground carbon (other parts of the carbon cycle).

#### Climate change mitigation outcomes

**Fig. 10:**
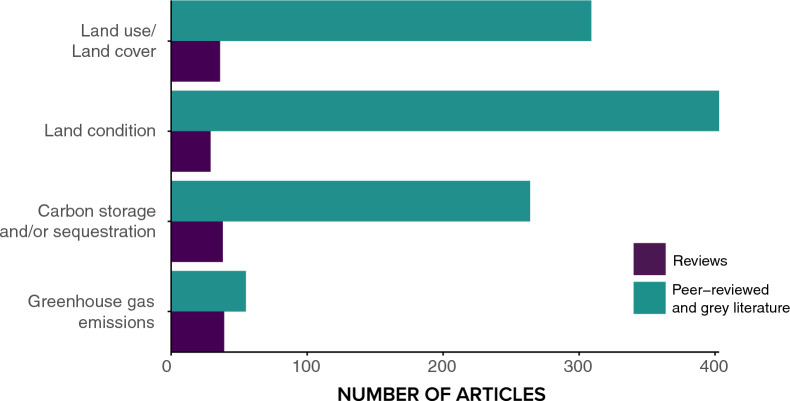
Distribution and frequency of climate change mitigation outcomes assessed within the evidence base. Articles can appear in more than one outcome type

Climate change mitigation outcomes were most frequently reported with proxy measures (changes to land condition (n = 403) and LULC (n = 309)), followed by changes to carbon storage and/or sequestration (n = 264). The least studied outcome was direct measures of changes to GHG emissions (n = 55) (Fig. 10).

Most articles only assessed one type of measure of climate change mitigation outcomes (n = 841, 88%). Articles which assessed more than one outcome, primarily examined changes to land condition and carbon storage and/or sequestration (n = 51 articles) with fewer looking at LULCC and change in carbon storage and/or sequestration (n = 15 articles).

#### Other outcomes assessed

The most well-studied other outcome of the articles examined in the systematic map was soil carbon storage and/or sequestration (n = 136; 14% of articles), followed by economic well-being (n = 53, 6%) and ecosystem function outcomes (n = 41, 6%) (Additional file 9: Fig. S9). In comparison, most review articles examined ecosystem function outcomes (n = 156; 16% of reviews), agricultural productivity (n = 107; 11%), and ecological community outcomes (n = 98; 11%). This implies that while there is likely significant research attention on the impact of NbIs on ecological and economic well-being (including productivity) individually, there is relatively less research attention on how these outcomes are or are not realized alongside climate change mitigation outcomes.

**Fig. 11:**
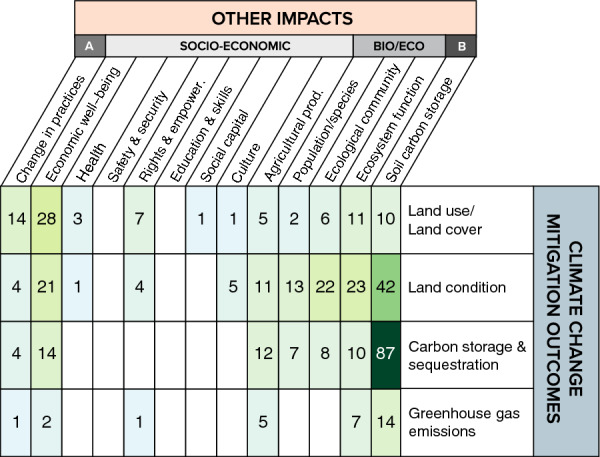
Heatmap of the occurrence of peer-reviewed articles and grey literature reporting climate change mitigation outcomes and other outcomes. **A** Adoption/update of land and/or agricultural management practices, **B** Belowground carbon storage and/or sequestration

We assessed how often articles evaluated core climate change mitigation outcomes as well as other outcomes (co-benefits, adoption/behavior change, belowground carbon). Overall, 31% of the evidence base measured both core climate change mitigation outcomes and other outcomes (n = 298 articles). Changes in soil carbon storage and/or sequestration were most commonly reported alongside changes in aboveground carbon storage and/or sequestration (n = 87 articles) and changes in land condition (n = 42 articles). Changes in ecological outcomes were reported to a lesser degree, while other types of social outcomes (aside from economic well-being) were not frequently reported (Fig. 11).

### Linkages between nature-based interventions and climate change mitigation outcomes

**Fig. 12:**
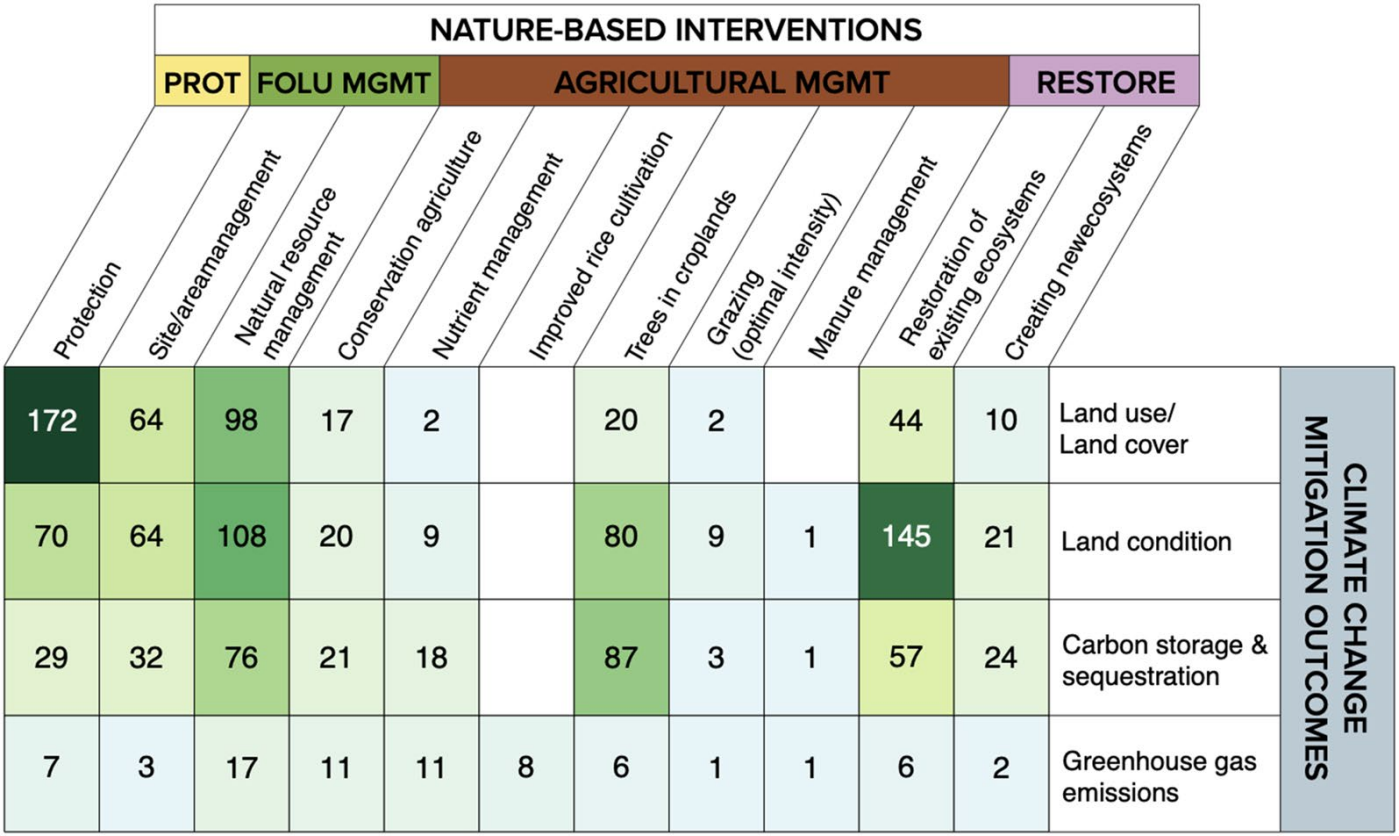
Heatmap of the distribution and occurrence of peer-reviewed and grey literature articles examining linkages between nature-based interventions and climate change mitigation outcomes. *PROT* protection, *FOLU* *MGMT* forest and other land use management, *AGRICULTURAL* *MGMT* agricultural management, *RESTORE* restoration. Articles can examine more than one linkage and may show up in more than one cell

A heatmap illustrates the distribution of articles examining linkages between NbIs and core climate change mitigation outcomes (Fig. 12). The most studied linkage is between protection-related interventions and LULCC outcomes (n = 172 articles, 18%). Linkages between restoration of existing ecosystem interventions and changes to land condition (n = 145 articles, 15%), natural resource management and changes to proxy outcomes (n = 92 articles, 10%), trees in croplands (e.g. agroforestry) and changes in land condition (n = 80 articles, 8%), as well as carbon storage/sequestration (n = 87 articles, 9%) were also well studied. However, there was a significant gap in terms of articles examining links between agricultural management (apart from trees in croplands) generally with both proxy and direct measures of mitigation (n = 77 articles, 8%). While changes in GHG emissions are the most proximate measurement of climate change mitigation, articles rarely reported outcomes in this area.

**Fig. 13:**
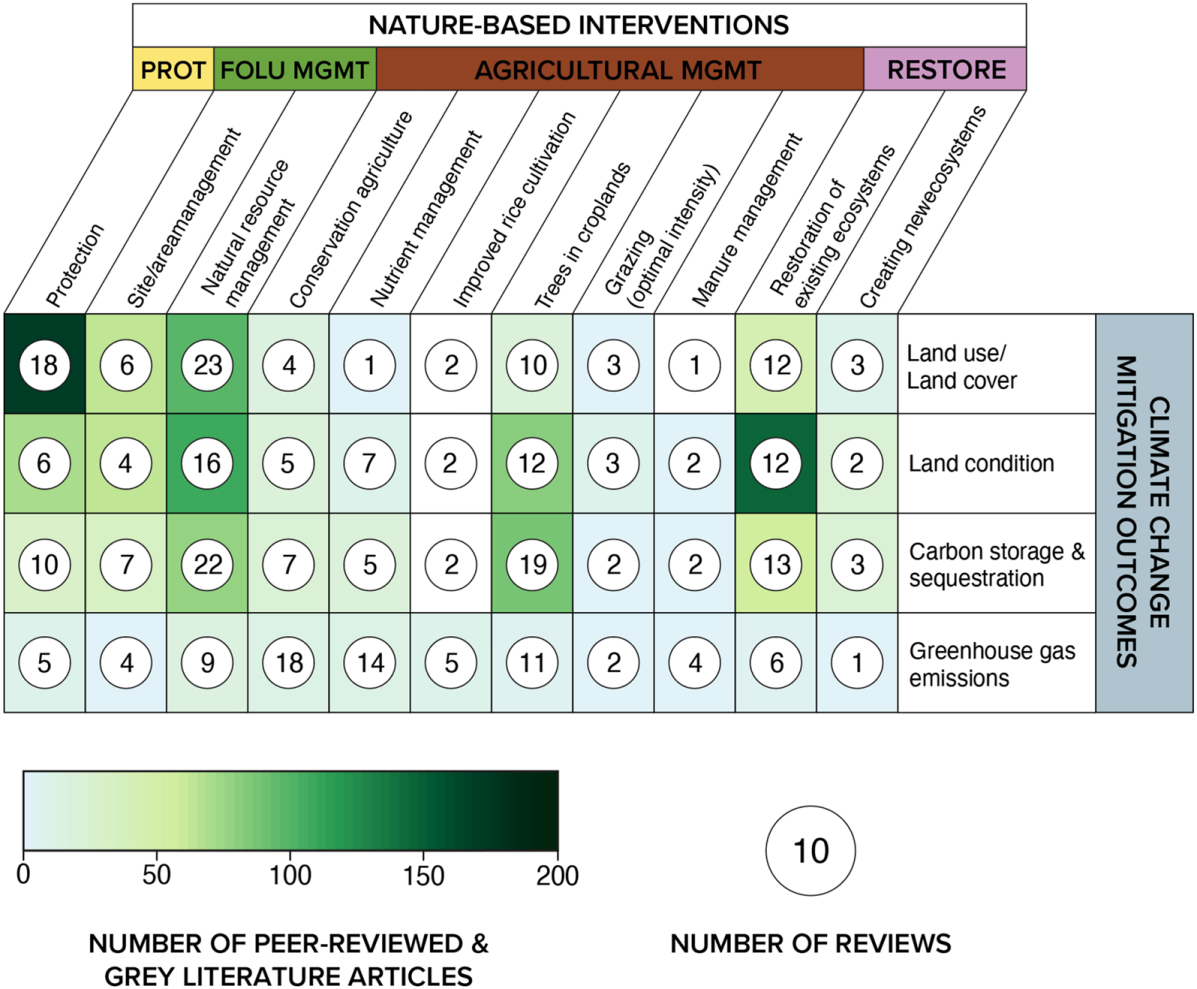
Distribution and occurrence of review articles (circles) in relation to peer-reviewed and grey literature articles, across linkages between nature-based interventions and climate change mitigation outcomes. *PROT* Protection, *FOLU* MGMT forest and other land use management, *AGRICULTURAL* *MGMT* agricultural management, *RESTORE* restoration

Comparatively, we find similar patterns in terms of the distribution of existing reviews which examine linkages between NbIs and climate change mitigation outcomes, indicating that areas of high volumes of evidence have likely already been reviewed (Fig. 13). Most reviews seek to synthesize the impacts of natural resource management interventions on LULCC (n = 23) and carbon storage and/or sequestration (n = 22). Interestingly, efforts to review the impacts of NbIs on GHG emissions far outstrip the evidence base in terms of recovered primary research studies and reports (Fig. 13). This is particularly the case for reviews on the links between agricultural management and restoration interventions and GHG emissions. This pattern may be due to the difference in geographical coverage of peer-reviewed and grey literature included in this systematic map versus the geographic focus of included reviews. Upon closer assessment, reviews on GHG emissions tended to be global in scope while this effort focuses on tropical and subtropical biomes—thus reviews tended to include studies that we would not have included in this map. For example, out of the 18 reviews on conservation agriculture and GHG emissions, 13 (72%) are global in scope. In addition, while there may be more reviews than primary research studies, this does not imply that reviews contain a significant volume of studies.

### Linkages between mitigation, socioeconomic, and biological/ecological outcomes

**Fig. 14:**
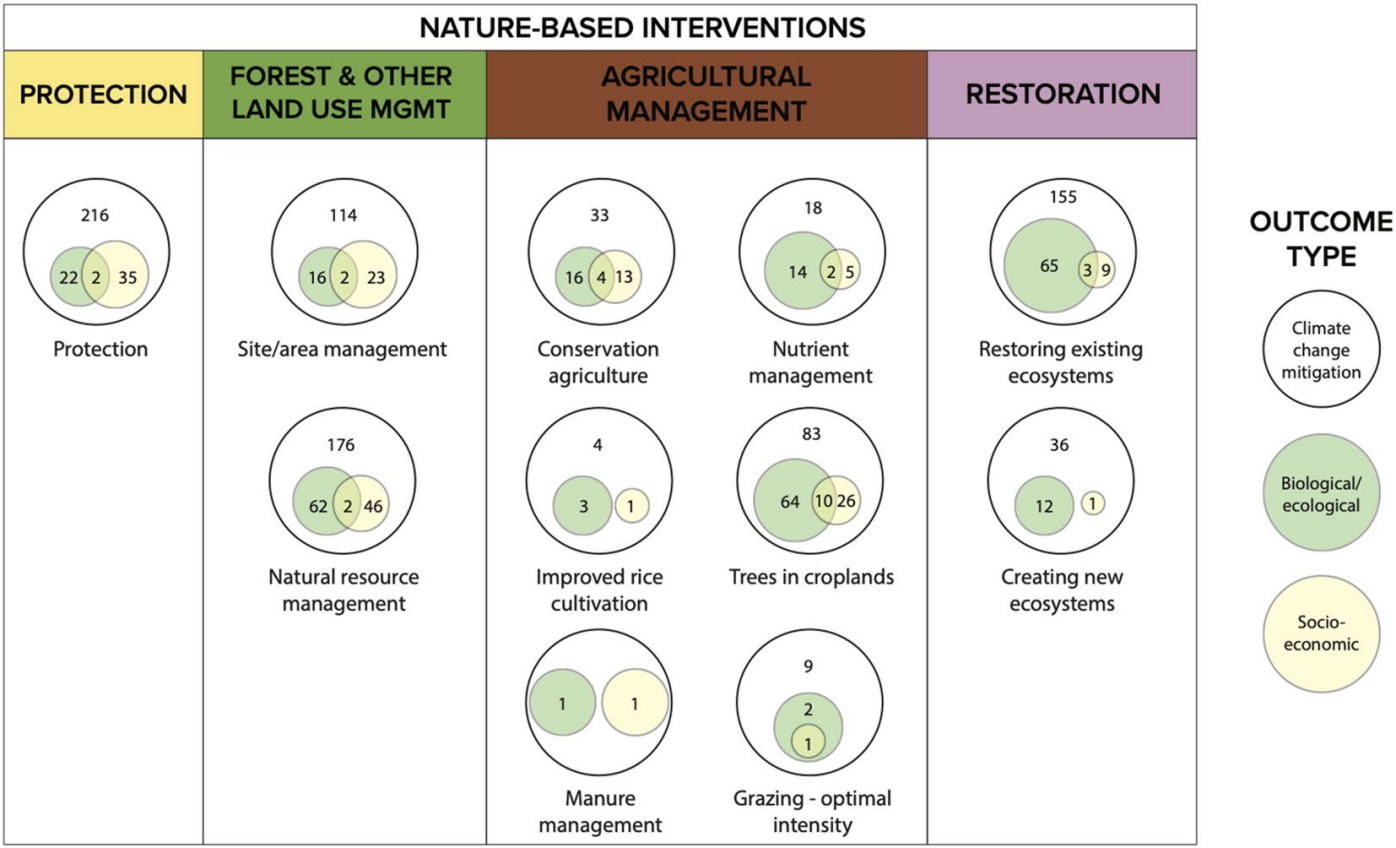
Distribution of articles that examined other outcomes across nature-based intervention types. Numbers within the white circles represent the total number of articles that only measured climate change mitigation outcomes. Numbers in the green circles represent those that measured biological and/or ecological outcomes (including belowground carbon storage and/or sequestration). Numbers in the yellow circles represent those that measured socioeconomic outcomes (including changes in adoption and/or uptake of land or agricultural management practices). Numbers in the overlap between circles represent the number of articles that measured all three outcome types

Generally, articles in the evidence base did not frequently examine other outcomes (n = 298, 32%) (Fig. 11) of NbIs. For agricultural management and restoration interventions, most articles examining other outcomes focused on biological/ecological in addition to climate change mitigation outcomes, with very few articles examining socioeconomic outcomes and just a handful focusing on all three (Fig. 14). Of the articles that did examine all three outcome types (for a given intervention, the most common combination was those measuring changes in proxy outcomes for climate change mitigation (core mitigation), ecosystem function (biological/ecological outcome), and agricultural productivity (socioeconomic outcome). For protection and forest and other land use management interventions, more articles measured socioeconomic outcomes than biological/ecological outcomes (Fig. 14). Comparing this with the distribution of existing reviews on co-benefits, we also see that only 25% of included reviews examined both climate change mitigation outcomes and either socioeconomic (n = 56 reviews), biological/ecological (n = 63 reviews), or all three outcomes (n = 40 reviews). We see significantly greater attention on reviewing links between NbIs and individual outcomes of ecological and socioeconomic responses (Additional file 9: Fig. S6). Overall, these findings illustrate that there is more research attention focused solely on impacts of interventions on ecological or social outcomes than research on how these outcomes vary alongside climate change mitigation outcomes. The overall evidence base on other outcomes within the context of climate change mitigation outcomes is significantly smaller—illustrating a potential evidence gap for understanding the broader systemic impacts of NbIs.

**Fig. 15:**
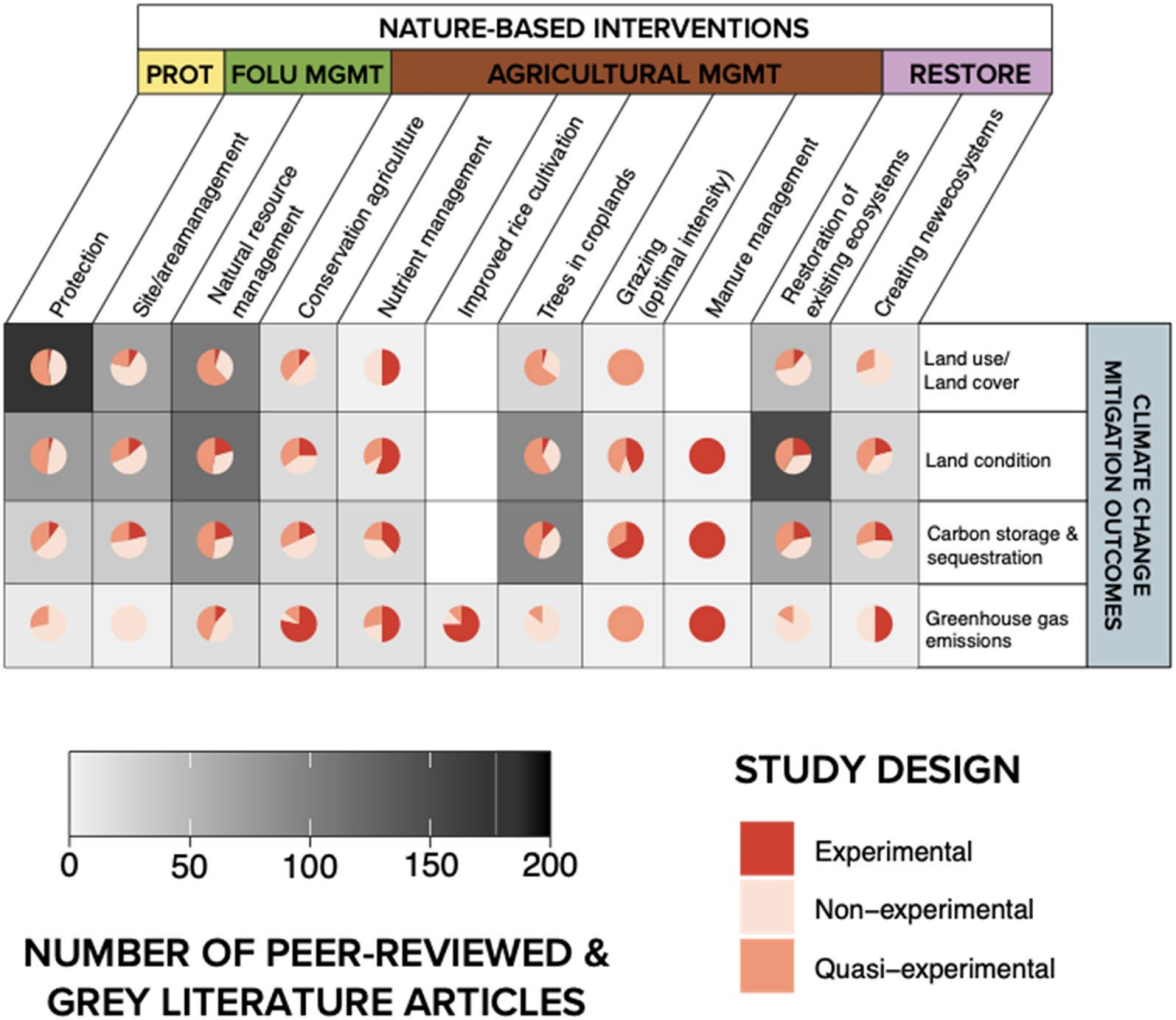
Distribution of study designs employed across linkages between nature-based interventions. *PROT* Protection, *FOLU* *MGMT* forest and other land use management, *AGRICULTURAL* *MGMT* agricultural management, *RESTORE* restoration (see Additional file 9: Table S3 for summary)

Lastly, this assessment disaggregated between types of study designs employed across peer-reviewed and grey literature articles in the evidence base. This was used as a heuristic illustration of what evidence exists for addressing questions of causal relationships between interventions and outcomes. Overall, we find that while there were fewer studies that examined changes to GHG emissions, most of the ones that did were experimental designs—for example, flux chamber experiments in agricultural management plots (e.g. [65–67]). However, this was not the case for protection and forest and other land use management interventions. Changes to LULCC tended to be assessed through non-experimental designs, except in the case of trees in croplands and natural resource management (Fig. 15). Changes to land condition tended to be measured with quasi-experimental designs while changes to carbon sequestration and storage were mixed across all study design types.

### Limitations of the map

While the search strategy employed in this map was intended to capture a comprehensive breadth of topics, it was not exhaustive, given available time and resources and thus may have resulted in a few limitations in assessing the evidence base. First, the search was limited to English, and we excluded a small volume of studies that were in Chinese, Spanish, Portuguese, and French. This highlights the potential for future expansion of this evidence base to explicitly search for and assess articles in other languages. Second, this map was limited to tropical and subtropical biomes and to aboveground ecosystems—but given the complexity of the carbon cycle and the diversity of ways that research refers to different types of ecosystems and interventions, there is a chance that some articles relevant to our search may have been missed. For example, we did not focus on belowground outcomes, and did not search explicitly for ecosystems where the majority of outcomes are likely belowground (e.g. wetland-type ecosystems and peat forests). However, our search results did include some related ecosystems such as flooded forests, peat forests, and mangroves. Thus, while we spent considerable time identifying and testing search terms, we recognize that this map may not be fully representative of these ecosystems.

In addition to limitations in our search strategy, we highlight a few potential caveats regarding the scope of our map and screening procedure that should be considered when interpreting this systematic map and using it to inform different types of decision. First, given the volume of articles that were returned from our searches, we relied on machine learning and natural language processing to help identify the most relevant articles for screening. To mitigate the risk of accidentally excluding relevant articles, we used a semi-automated process that retains user oversight for inclusion. We set a heuristic stopping point for screening when our rate of return on relevant articles for inclusion fell below 5 out of every 300 articles screened. Thus, it is possible that some relevant articles were missed in what we did not screen. However, the distribution of our results reflect other reviews in related topics e.g. [4, 24], and indicates that this systematic map is representative of the broad distribution of articles in the evidence base. Second, we did not assess the quality of study designs, but instead used the study design category as a heuristic for determining the current state of the evidence base and its ability to speak to causal mechanisms and inference in relation to the impacts of NbIs on mitigation outcomes and co-benefits. Third, we did not include articles that only measure changes in carbon storage and/or sequestration rates belowground (e.g. soil carbon). We recognize that this limits the ability of the evidence base to provide more comprehensive insight into the state of knowledge on the links between NbIs and impacts throughout the entire carbon cycle. Future reviews could expand this evidence map by looking more explicitly at belowground carbon outcomes.

## Conclusions

This systematic map and accompanying map of reviews highlights several areas where there is substantial research attention, as well as evidence ‘gaps.’ Generally, areas of substantial research attention also had several existing reviews (including systematic reviews, meta-analyses, and other well-documented reviews), suggesting that sufficient resources exist that summarize existing knowledge. However, this map also identified broader evidence gaps—particularly in the relatively lower levels of research attention on the links between NbIs and direct measures of climate change mitigation outcomes (i.e. changes to aboveground carbon storage and/or sequestration, GHG emissions) in comparison to measured changes to proxy outcomes (i.e. land cover, use, condition) as a whole, and much broader and deeper gaps on the impacts on social and/or ecological outcomes. While some interventions have significant volumes of studies, including studies performed at nearly global or pantropical scales, most research tends to focus on local scale impacts. Across the evidence base, relatively few studies examined how contextual factors and aspects of project design and implementation may mediate the impacts of NbIs. In addition, this map reveals poor reporting of data on costs of NbIs—limiting abilities to better determine cost-effectiveness of different interventions to inform future planning, particularly in anticipating intended and unintended consequences. Overall, the systematic map suggests that the existing evidence base confers a moderate ability to better understand to what extent and how protection, restoration, and sustainable management interventions have an impact on climate change mitigation and associated benefits to people and nature.

## Implications for research

### Focus on proxy measurements of mitigation

The value of NbIs for climate change mitigation depends critically on their ability to contribute to increasing carbon storage and/or avoiding GHG emissions. Therefore, building a solid evidence base on the impact of these interventions requires an accurate quantification of changes to carbon storage, sequestration, and GHG emissions. Overall, the results demonstrate that NbIs mitigation outcomes are primarily measured in terms of indirect outcomes (e.g. LULCC and land condition) (measured by 675 articles, 56%) and less often use direct mitigation measures (e.g. carbon storage and/or sequestration and GHG emissions) (measured by 33% of articles). The continued reliance on proxy measures suggests large evidence gaps remain underlying the additionality of NbIs, i.e. their ability to mitigate GHG emissions beyond that expected in the absence of interventions. While the use of—and extrapolation from—allometric equations models could advance our understanding of the carbon mitigation potential of different land stewardship interventions, we note that the estimates are not exempt from uncertainties and particularly in relation to tropical landscapes [34, 68, 69]. While the use of recent technologies such as Light and Radio Detecting and Ranging (LIDAR and RADAR) have allowed the upscaling of carbon stock assessment, as well as improved the measurement of carbon fluxes (e.g. through using Eddy covariance flux towers), they can often be cost-prohibitive at scale. Thus, determining cost-effective ways to fill this evidence gap, e.g. using harmonized and validated high-resolution carbon density maps [70], will be critical for understanding the relative net impacts of different interventions [4].

### Available evidence to support causal inference

The ability to accurately assess whether and how NbIs contribute to climate change mitigation impacts relies upon credible studies (e.g. well-designed experimental and quasi-experimental approaches [71] that can support causal inference. Overall, we note that more than half of included articles (57%) employ comparative approaches for impact evaluation (including the use of quasi-experimental and experimental research designs) demonstrates that these methods have been increasingly mainstreamed in the evaluation of NbIs effectiveness. While this result indicates that there is potentially sufficient volumes of evidence that can be used to assess the magnitude and direction of the effect size of at least some interventions, we offer a couple of caveats for interpretation.

While many articles employed quasi-experimental and experimental approaches, we cannot speak to whether each experimental and quasi-experimental study was sufficiently well-designed to support any claims of causal attribution and unbiased estimation of effectiveness. As this is a systematic map, we do not critically appraise the quality of study designs and recognize that studies which compare, for example, outcomes before and after an intervention, or between intervention and non-intervention sites, may be less robust for causal attribution given the potential for statistical bias and unaccounted confounding factors. As any study design that employed a control for comparison (before/after, with/without) was considered “quasi-experimental,” the large volume of studies should not be interpreted as an indicator of study quality for causal inference.

Articles using experimental methods most often employed in vitro tests (e.g. experimental plots) to test intervention design, rather than experimentally testing impacts of in vivo interventions (e.g. randomized controlled trials), particularly for studies on agricultural management and restoration. While in vitro experiments can be rigorous tests of the effects of specific actions on land cover, land condition and associated climate change mitigation outcomes, testing the performance of interventions in real-life conditions, for example—at higher scale or as part of an intervention lead by an organization, would allow better understanding of the performance of NbIs in more complex contexts. More coordinated efforts are nevertheless needed to better and more rigorously investigate the various mechanisms and heterogeneous impacts of most NbIs across different implementation contexts.

### Lack of attention on broader socio-ecological outcomes

Consideration of other social and ecological impacts of NbIs in project planning, implementation, and evaluation are increasingly important to national governments and bilateral and multilateral donors as they define and implement relevant policies. While the map of reviews demonstrates that research attention on the social and ecological impacts linked to NbIs is high (e.g. [4, 46, 82]) more holistic, systemic examinations of direct measures of mitigation and alongside other social and ecological impacts is a significant evidence gap. This is particularly critical for areas such as safety and security where there is a complete evidence gap despite widespread assumptions that mitigation outcomes can be achieved alongside improved resilience to environmental shocks and stresses (an example of security).

This gap may exist for a few reasons. First, it takes a significant amount of resources (money and time) to truly study climate change mitigation from a broader social-ecological systems perspective, and these resources for interdisciplinary study are currently limited (e.g. [83]). Budgets for project monitoring and evaluation are often insufficient to enable the collection of adequate data and implementation of study designs that can test assumptions and make causal inferences for impact (e.g. [84, 85]). Second, the scale and scope of research and action are often misaligned—leading to breakdowns in knowledge transfer and uptake (e.g. [86]). While all respective communities try to study broadly the same research questions related to climate change mitigation, they do not always measure it using the same metrics or tools, thereby limiting the transfer of knowledge from one discipline to another and providing an incomplete picture to practitioners, decision-makers, and the public. Third, this lack of attention may also reflect the difficulties in achieving “win-wins” across multiple outcomes [84, 87, 88] and bias in reporting only success stories versus trade-offs and/or failures [62, 89]. More importantly, there are also disconnects and challenges in aligning research questions with data required to inform performance indicators or decisions and priorities for local stakeholders. In addition, there are often practical challenges for collection of long-term and multi-disciplinary data, like the fact that impacts occur at different timescales (e.g. [90, 91]). Overall, these factors result in the ongoing proliferation of datasets that are suited for specific purposes, but are challenging to aggregate and synthesize across temporal and spatial scales to better understand overall trends in effectiveness.

### Priorities for ongoing and future research

This evidence map highlights critical evidence gaps in the following areas which should be prioritized for future research: (1) measurements of realized change in carbon storage, sequestration, and GHG emissions; (2) assessments of causal impacts across scales; and (3) research attention on impacts within broader socio-ecological systems. Given the multitude of likely reasons for these gaps—the findings from this map have implications both for increasing and diversifying research attention as well as fostering improved coordination and collaboration across disciplines and sections. Our ability to scientifically understand how (heterogeneous) impacts are affected by enabling conditions, mechanisms, and design features relies on integrating systems-thinking approaches to better articulate assumptions [45, 92], concerted efforts to collect data across scales and conduct transdisciplinary analysis, and increased deployment of study designs that can test causal relationships (e.g. [93, 94]).

This evidence map illustrates that while substantial information exists to better inform the calculation of carbon sequestration and avoided emissions potential of different types of ecosystems and from different types of interventions, this information is biased towards specific ecosystems (mostly forests) and interventions (primarily protection). Similarly, there are also biases in the evidence base towards proxy measures for mitigation, and further research that links changes in these proxies with accompanying changes for storage, sequestration, and/or avoided emissions is urgently needed. These trends also highlight the need for more broad, comparative meta-analysis of the mitigation effect sizes of different types of interventions across different contexts, as opposed to more narrow meta-analyses that are generally limited to only one or a few conservation, restoration, or sustainable land management interventions. To facilitate these meta-analyses, the evidence base also needs to be improved with additional, intentional, and harmonized research designs which can help fill identified knowledge gaps on the mitigation outcomes for specific interventions in various contexts.

Theory-based evaluation allows us to ensure we understand how conservation fits within existing social-ecological systems and how they may effect change [95]. While examination and evaluation of systemic change is needed to understand which variables can affect both the implementation and impacts of various types of interventions across different contexts, this requires trans- and interdisciplinary approaches. In particular, coordinated data collection across contexts and disciplines can help better inform predictions about how NbIs will fare under different climate change scenarios [96] and how they can deliver on future social, ecological, and climate objectives [97]. The findings from this map suggest that improving the evidence base will require the following:

1. Increased research effort on systemic change: this includes research that robustly examines the causal linkages between proxy outcomes (e.g. land condition, LULCC) and mitigation outcomes, as well as research on linked changes across social, ecological, and climate outcomes.
2. Stronger collaborations between social and environmental scientists: these collaborations need to be adequately incentivized and resourced [98]—both through institutional and financial support and incentives as well as through the development and use of cross-disciplinary tools which can track various dimensions of conditions that foster the longevity of environmental projects (e.g. governance and management—Elinordata.org [99]. Increased incentives to fund long term monitoring and evaluation, and to learn from failure, are needed to generate an evidence base that can inform practice over time [100].

## Implications for policy/management

### Gaps in reporting cost information

Overall, reporting of cost information was poor across the evidence base, particularly from peer-reviewed articles. Comparatively, cost data was more frequently reported, and reported in greater detail, in grey literature reports and articles—potentially because cost data may be more relevant for grey literature audiences, along with fewer publishing constraints. This evidence gap reflects findings of poor cost reporting across conservation and development (e.g. [72–76]). In this assessment, costs were most frequently reported for payments or incentives (e.g. PES) within protection and management interventions (n = 12 and 9 articles, respectively). In some cases, these articles examined costs of payments/incentives within the context of understanding the process and outcomes of determining where and how to distribute payments across households, communities, and locations (e.g. [77]). However, other types of costs (implementation, opportunity, monitoring, and evaluation, operational) that are equally important were not frequently reported [78]. The overall lack of cost reporting is a major limitation for comprehensively and accurately understanding the realized cost-effectiveness of different types of interventions. Consideration of cost data has likely benefits—for example—identifying efficiency gains and assessing tradeoffs between costs and desired objectives to inform decision-making [79, 80]. However, doing so will require both robust data on impacts for climate change mitigation, nature, and people objectives as well as detailed data on a wide range of costs in order to identify areas with potential for high return on investment [81].

### Priorities for ongoing and future practice and policy

The trends in the evidence base have a few key implications for policy and practice related to NbIs. Without a rigorous and systematic evidence base, not only do we not have a clear idea of what interventions are effective for achieving the goals outlined under the Paris Climate Agreement and other major international agreements, conventions, and frameworks—but we lack information on how these outcomes, if achieved, can be sustained over time.

NbIs are not new—they have been implemented for decades throughout conservation, environmental, and natural resource management. Thus, this lack of rigorous and systematic published evidence on other broader impacts (including social and ecological) and mitigation has two implications for policy-makers and practitioners in this space. First, and foremost, one of the challenges in compiling and assessing the evidence base on NbIs was the lack of agreed upon terminology regarding these interventions. Oftentimes, definitions for intervention types are conflicting or overlapping, and in practice, are difficult to operationalize when examining existing knowledge from before these new “concepts” were mainstream. Second, the current monitoring and evaluation systems are insufficient to collect, collate, and share necessary data to inform adaptive management, strategy, and future implementation. Part of this insufficiency may be due to temporal lags and misalignments between organizational and research agendas, resulting in a disconnect between research and action (e.g. [101, 102]). In order to build a sufficient, dynamic, and relevant evidence base to support NbI decision-making we recommend the following:

1. Partnerships across implementation organizations and bilateral and multilateral donors to coordinate shared frameworks and processes: Formalized and resourced partnerships to coordinate collection, collation, and sharing data is needed to facilitate efficient and inclusive planning and design, as well as evaluations of impacts within and across scales. This is particularly critical as the evidence base demonstrates that significant volumes of potentially relevant information exist, but may be not sufficiently explored. While there are some emergent efforts (e.g. World Bank ClimateWarehouse, Nature4Climate), they have not been widely adopted nor populated with data.
2. Shared and enforced rules for transparency and accountability with data sharing and use: These partnerships not only need to be supported through long-term funding that mandates transparency and accountability, but they need to come with an agreed upon set of rules of sharing sensitive information and regulating use. Monitoring, evaluation, and learning frameworks should be co-designed with local actors, particularly in areas managed by Local Communities and/or Indigenous Peoples, through a participatory and inclusive process. In addition, conservation organizations should work to improve and build capacity to collect, use, and manage this data to support adaptive management and knowledge sharing—and collectively improve and augment the evidence base.
3. Actors engaged with and in carbon markets should leverage the evidence base and consider gaps when estimating the likelihood and reliability of estimates for payment schemes: In particular, these actors should ensure that cost data is incorporated into offset estimation in transparent ways to better assess risks, uncertainty, and cost-effectiveness of NbI implementation in different contexts and under different scenarios.

The evidence base demonstrates that potentially robust information exists on some NbIs and their links to mitigation outcomes, however, the distribution and characteristics of existing evidence are biased towards certain interventions, geographies, and may lack reliability for supporting causal attribution. Thus, further developing the evidence base requires several key and critical actions. Well-resourced and institutionally-supported coordination and sharing of data and information, and collaborative evaluations and learning are needed to accurately inform the planning, implementation, and monitoring of NbIs. Long-term impact evaluations are needed to evaluate the impacts of NbIs as well as to better understand how to balance gains and tradeoffs in the short and long-term for mitigation and sustainability goals. Finally, research on pathways and mechanisms that drive changes in social, ecological, and climate outcomes from NbIs is urgently needed if we are to make progress towards global goals for sustainable development, biodiversity conservation, and climate change mitigation.

## Supplementary Information

**Additional file 1. **Protocol for map of reviews.

**Additional file 2. **Search strategy.

**Additional file 3. **Grey literature search strategy and record.

**Additional file 4. **Intervention typology.

**Additional file 5. **Outcome typology.

**Additional file 6. **Coded metadata from primary research.

**Additional file 7. **List of included articles (for map of primary research and map of reviews).

**Additional file 8. **List of excluded articles at full text for systematic map.

**Additional file 9: Supplementary Figures and Tables Figure S1.** ROSES flow diagram for map of reviews. **Figure S2.** Growth in peer-reviewed publications, grey literature, and reviews since 1990. **Figure S3. **Affiliation type of first authors of included articles. **Figure S4. **Types of comparators employed in peer-reviewed articles and grey literature across different types of outcomes measured and disaggregated by study design. **Figure S5. **Geographic scale of study of articles within systematic map and map of reviews. **Figure S6. **Distribution and occurrence of review articles examining links between nature-based interventions and co-impacts. Reviews included did not have to focus on links to climate change mitigation outcomes. **Figure S7. **Distribution and frequency of complementary actions implemented alongside nature-based interventions to address enabling conditions. Articles can appear in more than one complementary action type. **Figure S8. **Number of articles in the evidence map reporting cost information disaggregated by publication year. **Figure S9**: Distribution and frequency of articles measuring changes to co-benefits assessed within nature-based interventions. Articles can appear in more than one co-benefit type. **Table S1. **Distribution of categories of nature-based interventions examined across biome types in the systematic map. Articles can examine more than one pathway type and more than one biome. **Table S2. **Distribution of categories of nature-based interventions examined by geographic region in the systematic map. Articles can examine more than one pathway type and more than one region. **Table S3.** Distribution of included articles across intervention and outcome linkages, disaggregated by study design type (complementary to Fig. 15).

**Additional file 10. **Coded metadata from reviews.

**Additional file 11. **ROSES Reporting Forms for systematic map.

**Additional file 12. **ROSES Reporting Form for map of reviews.

## Data Availability

The datasets generated and/or analysed in this study are available in the supplemental materials.
