# Supplementary material for: Assessing evidence on the impacts of nature-based interventions for climate change mitigation: a systematic map of primary and secondary research from subtropical and tropical terrestrial regions
Source: Environ Evid. 2023 Oct 25;12:21. doi: 10.1186/s13750-023-00312-3 (PMC11378798; doi:10.1186/s13750-023-00312-3)
Supplement: Supplementary file 1 — Additional file 1. Protocol for map of reviews. [file 13750_2023_312_MOESM1_ESM.docx]

**Additional File 1 -** Protocol for Map of Reviews

This file provides the detailed inclusion criteria for the map of reviews along with the process to assess review quality.

**Map of Reviews Inclusion Criteria and Screening Process**

**Objective of the map of reviews:**

This study is a rapid effort to systematically map and describe the distribution of existing reviews and meta-analyses on the links between nature-based interventions for climate change mitigation and socio-economic and biophysical outcomes. This is complementary to a systematic map that identifies, maps, and describes the evidence on the impacts of nature-based interventions on climate change mitigation outcomes - including reviews. The objective of this map of reviews is to characterize the existing synthesized evidence base on socio-economic, biological, and ecological impacts in order to provide context for the findings of the main systematic mapping effort that is focused on impacts on (1) land and forest management practices and restoration practices (2) climate change mitigation outcomes in forest, agricultural, and grassland landscapes in tropical regions.

**Overarching Key Question:**

What is the evidence base for the links between nature-based solutions interventions and impacts on socio-economic and biophysical outcomes in forest, agricultural, and grassland landscapes in tropical regions?

**Elements of the Primary Question:**

**Population**

- Terrestrial ecosystems and mangroves in tropical countries

**Intervention**

- Land stewardship interventions that aim to protect, manage, or restore existing natural terrestrial ecosystems
- Land stewardship interventions that aim to create or manage new ecosystems in non-urban/peri-urban areas
- Interventions that aim to promote and implement sustainable and/or climate-smart agriculture, grazing, and agroforestry management and practices.
- Studies covering passive restoration and conservation interventions

**Study type**

- Systematic maps and reviews, gap maps, or any literature review that clearly documents the search strategy and provides a list of included articles. Review articles must fulfill the following criteria (derived from CEEDER, Konno et al. 2020):
  - (i) Provide details on specific databases, search engines and/or organizational websites searched;
  - (ii) List the search terms used; and
  - (iii) Include a separate list of articles included in the analysis.
- Review articles that aim to examine the link between an NCS intervention and one or more of the target outcomes

**Outcomes**

- Socio-economic outcomes (including provisioning and cultural ecosystem services)
- Biological and ecological outcomes (including regulating and maintenance services)
- Change in land and/or agricultural practices or knowledge gain

**Assessing review quality**

We did not conduct full CEEDER scoring for all reviews, instead we used three reporting requirements from CEEDER (Konno et al. 2020) for inclusion as a heuristic of minimum review reporting quality. Specifically, reviews must (i) provide details on specific databases, search engines and/or organizational websites searched; (ii) list the search terms used; and (iii) include a separate list of articles included in the analysis.
