# Supplementary material for: Assessing evidence on the impacts of nature-based interventions for climate change mitigation: a systematic map of primary and secondary research from subtropical and tropical terrestrial regions
Source: Environ Evid. 2023 Oct 25;12:21. doi: 10.1186/s13750-023-00312-3 (PMC11378798; doi:10.1186/s13750-023-00312-3)
Supplement: Supplementary file 2 — Additional file 2. Search strategy. [file 13750_2023_312_MOESM2_ESM.docx]

**Additional File 2 - Search Strategy**

This file provides the comprehensive search methods for our systematic map.

The methodology for building this systematic map included three phases: (1) establishing key search terms to capture the concepts identified in the conceptual framework; (2) developing the scope and strategy of the search and inclusion/exclusion criteria; and (3) developing the scope and extent of a coding framework to extract relevant information to characterize the evidence base.

*Searches*

A comprehensive search was undertaken to best capture an unbiased representation of existing literature related to our research question.

*Language*

Given available resources for this systematic map, we only search in English. We recognize that this may bias our systematic map as there may potentially be relevant literature in other languages [50].

*Search terms*

We compiled a set of search terms relevant to different components of the research question. This string of terms was scoped in the Web of Science Core Collection to examine sensitivity given alternate terms and wildcards, as well as using input from our stakeholder team.

*Population*

(forest OR woodland OR meadow OR pasture OR agricultur* OR rangeland OR grassland OR mangrove OR tree OR cropland OR grazing OR land OR ecosystem OR landscape OR rice OR tropic*)

**AND**

*Intervention*

(restoration OR reforestation OR afforestation OR replanting OR rehabilitation OR enrichment OR "tree islands") OR ("rice production" OR "rice intensification" OR "rice cultivation" OR "community forest" OR "community forests" OR "community forestry" OR "shade grown" OR "climate-smart" OR "pasture management" OR "cover crop" OR "cover crops" OR "nutrient management" OR agroforestry OR agroforest OR silvopastor* OR silvopastur* OR silvo-pastor* OR silvo-pastur* OR agro-ecolog* OR agroecolog* OR "conservation agriculture" OR "tree planting" OR fencing OR exclosure OR ((partial OR selecti* OR gap OR retention) NEAR/3 (felling OR cutting OR harvest*)) OR "grazing management" OR "active management" OR "salvage logging" OR "reduced-impact logging" OR "alley cropping" OR "fire management" OR plantation OR "forest management" OR "manure management" OR ((crop OR cropland) NEAR/2 management) OR windbreaks OR thinning) OR ("protected area" OR "protected areas" OR ("Indigenous Peoples" OR "Indigenous communities" OR "Indigenous groups") OR "national park" OR "concession" OR "buffer zone" OR "sacred groves" OR "sacred forests" OR "sacred forest" OR "sacred grove" OR (protection NEAR/2 (forest OR landscape OR grassland))) OR ("land stewardship" OR "natural climate solutions" OR "natural climate solution" OR "ecosystem-based adaptation" OR "carbon forestry" OR "payments for ecosystem services" OR "payments for environmental services" OR "PES" OR "REDD" OR "REDD+" OR "Reduced Emissions from Deforestation and Degradation" OR "sloping land conversion" OR "cropland to forest")

**AND**

*Outcome*

("land use change" OR "land-use change" OR "land conversion" OR "forest conversion" OR "grassland conversion" OR deforestation OR "land cover" OR "forest cover" OR "vegetation cover" OR "habitat cover" OR "tree cover" OR (clearing NEAR/4 (forest OR land)) OR ((diversity OR composition OR recovery OR succession) NEAR/1 (tree OR forest)) OR ((biomass OR biomasses) NEAR/2 (tree OR shrub OR woody OR aboveground OR above-ground OR recovery OR living)) OR (degradation NEAR/2 (forest OR grassland)) OR ((climate OR carbon OR CO2 OR GHG OR "greenhouse gas") NEAR/3 mitigat*) OR ((carbon OR CO2) NEAR/2 (sequestration OR balance OR accounting OR storage OR emission OR sink OR stock OR fixation OR density)) OR (("greenhouse gas" OR GHG) NEAR/2 (emission OR avoid* OR reduc*)) OR aboveground OR above-ground)

**AND**

*Outcome adjacent*

(impact OR effect* OR evaluat* OR empiric* OR assess*)

**NOT**

(Canada OR "British Columbia" OR Europe OR Sweden OR Norway OR Finland OR Scandinavia* OR Mediterranean OR Chile OR "United Kingdom" OR Korea OR Pakistan OR Russia OR Denmark OR England OR Wales OR Ireland OR Scotland OR "integrated water resource management" OR European OR Spain OR Spanish OR Alabama OR Alaska OR Arizona OR Arkansas OR California OR Colorado OR Connecticut OR Delaware OR Florida OR Georgia OR Idaho OR Illinois OR Indiana OR Iowa OR Kansas OR Kentucky OR Louisiana OR Maine OR Maryland OR Massachusetts OR Michigan OR Minnesota OR Mississippi OR Missouri OR Montana OR Nebraska OR Nevada OR "New Hampshire" OR "New Jersey" OR "New Mexico" OR "New York" OR "North Carolina" OR "North Dakota" OR Ohio OR Oklahoma OR Oregon OR Pennsylvania OR "Rhode Island" OR "South Carolina" OR "South Dakota" OR Tennessee OR Texas OR Utah OR Vermont OR Virginia OR Washington OR "West Virginia" OR Wisconsin OR Wyoming OR "north america" OR "north american" OR Albania OR Andorra OR Armenia OR Austria OR Azerbaijan OR Belarus OR Belgium OR Bosnia and Herzegovina OR Bulgaria OR Croatia OR Cyprus OR Czechia OR Estonia OR France OR Germany OR Greece OR Hungary OR Iceland OR Italy OR Kazakhstan OR Kosovo OR Latvia OR Liechtenstein OR Lithuania OR Luxembourg OR Malta OR Moldova OR Monaco OR Montenegro OR Netherlands OR Poland OR Portugal OR Romania OR Russia OR San Marino OR Serbia OR Slovakia OR Slovenia OR Switzerland OR Turkey OR Ukraine OR "Vatican City" OR Alberta OR "British Columbia" OR Manitoba OR "New Brunswick" OR Newfoundland OR "Northwest Territories" OR "Nova Scotia" OR Nunavut OR Ontario OR "Prince Edward Island" OR Quebec OR Saskatchewan OR Yukon OR Labrador)

**NOT**

TI=("modelling" OR "modeling")

**Comprehensiveness of the search**

We tested the comprehensiveness of the search string by testing it against a library of 30 relevant articles compiled by the review and stakeholder team and from backwards citation chasing. We refined the search terms until we recovered all items in the test library that were indexed in Web of Science. We explicitly tested the impact of using NOT terms on the comprehensiveness of our search. With careful development and testing, the use of NOTs can improve the efficiency and precision of a search strategy, particularly when dealing with large corpora (e.g. [51–53]). We tested the likelihood that the use of NOTs would result in a significant portion of missed citations using a random sample and a relevance ranked sample of studies (n=500 citations) that would have not been captured in a search strategy including NOT terms. We found that <1% of recovered search results were ultimately relevant. While this percentage is very low, we account for potentially missed studies by searching other publication databases, using backward citation chasing, as well as an extensive grey literature search.

**Searching the literature**

Search for relevant published and unpublished literature, including both peer-reviewed and grey reports, conducted from the following sources: (1) within bibliographies of relevant reviews; (2) online publication databases, and (3) organization websites and repositories. This systematic map will begin with a rapid search for relevant reviews within the scope of the research question that document and report their search strategy and provide a list of included articles in their review. These reviews were the basis for backwards citation chasing as a search strategy for identifying an initial pool of potentially relevant sources (see Supplementary Information 2 for protocol). Citation chasing is a search approach that uses a list of relevant studies as the source within which to identify relevant articles. *Backwards citation chasing* involves looking at the citations that relevant articles cite in their work and screening *those* citations for inclusion in this synthesis. Starting a search strategy using backwards citation chasing allows one to identify an initial evidence base that is more likely to be relevant. This can help refine methods and train machine learning models to sort through search results more quickly and efficiently during the screening process. Specifically, we conduct the following searches:

1. *Backwards citation searches* This map includes a subset of data specifically pertaining to the review question derived from the bibliographies of 39 relevant reviews identified through systematic searches in publication databases.
2. *Publication database searches* We searched Web of Science (Core Collection 1900-Present, 10 indices), Environment Complete (EBSCO). Selection of these databases was based upon previous systematic evidence syntheses on related topics (e.g. [20,54]). We searched these databases for studies published prior to 2021.
3. *Topical databases and organization searches* Targeted searches of specialist websites and databases were conducted, in particular, of established online repositories of impact evaluations, project and program evaluations, and systematic evidence syntheses on related topics to our research question. The first 100 results were screened per website/database.
4. *Stakeholders and topic experts* Key informants and the broader research community were contacted for relevant literature for screening and inclusion through electronic means (e.g. email, social media).

**Indices and databases searched include:**

**EBSCO (1900-present): Environment Complete**

**Web of Science (1900-present):** Science citation index expanded, Social sciences citation index, Arts & humanities citation index, Conference proceedings citation index. Science, Conference proceedings citation index. Social science & humanities, Book citation index. Science, Book citation index. Social sciences & humanities, Current chemical reactions, Index chemicus.

**Search results**

| **Database** | **Date searched** | **Search String** | **Results** |
| --- | --- | --- | --- |
| Agris | 1/21/2022 | (forest OR woodland OR meadow OR pasture OR agricultur* OR rangeland OR grassland OR mangrove OR tree OR cropland OR grazing OR land OR ecosystem OR landscape OR rice OR tropic*)AND"land stewardship" OR "natural climate solutions" OR "ecosystem-based adaptation" OR "carbon forestry" OR "payments for ecosystem services" OR "payments for environmental services" OR "PES" OR "REDD" OR "Reduced Emissions from Deforestation and Degradation" OR "sloping land conversion" OR "cropland to forest" OR "protected area" OR "indigenous peoples" OR "indigenous communities" OR "indigenous groups" OR "community-based conservation area" OR "national park" OR "concession" OR "buffer zone" OR "sacred forest" OR "sacred grove" OR "forest protection" OR "landscape protection" OR "grassland protection"AND"land use change" OR "land-use change" OR "land conversion" OR "forest conversion" OR "grassland conversion" OR "deforestation" OR "land cover" OR "forest cover" OR "vegetation cover" OR "habitat cover" OR "tree cover" OR "forest clearing" OR "land clearing" OR "diversity" OR "composition" OR "recovery" OR "succession" OR "biomass" OR "biomasses" OR "degradation" OR "climate mitigation" OR "climate change mitigation" OR "carbon dioxide mitigation" OR "CO2 mitigation" OR "GHG mitigation" OR "greenhouse gas mitigation" OR "carbon sequestration" OR "CO2 sequestration" OR "carbon stock" OR "carbon storage" OR "carbon balance" OR "carbon accounting" OR "carbon emissions" OR "CO2 emissions" OR "carbon sink" OR "carbon fixation" OR "carbon density" OR "greenhouse gas emission" OR "GHG emission" OR "aboveground" OR "above-ground"AND(impact OR effect* OR evaluat* OR empiric* OR assess*)NOT | 3 |
| Agris | 1/21/2022 | (forest OR woodland OR meadow OR pasture OR agricultur* OR rangeland OR grassland OR mangrove OR tree OR cropland OR grazing OR land OR ecosystem OR landscape OR rice OR tropic*)AND"land stewardship" OR "natural climate solutions" OR "ecosystem-based adaptation" OR "carbon forestry" OR "payments for ecosystem services" OR "payments for environmental services" OR "PES" OR "REDD" OR "Reduced Emissions from Deforestation and Degradation" OR "sloping land conversion" OR "cropland to forest" OR "protected area" OR "indigenous peoples" OR "indigenous communities" OR "indigenous groups" OR "community-based conservation area" OR "national park" OR "concession" OR "buffer zone" OR "sacred forest" OR "sacred grove" OR "forest protection" OR "landscape protection" OR "grassland protection"AND"land use change" OR "land-use change" OR "land conversion" OR "forest conversion" OR "grassland conversion" OR "deforestation" OR "land cover" OR "forest cover" OR "vegetation cover" OR "habitat cover" OR "tree cover" OR "forest clearing" OR "land clearing" OR "diversity" OR "composition" OR "recovery" OR "succession" OR "biomass" OR "biomasses" OR "degradation" OR "climate mitigation" OR "climate change mitigation" OR "carbon dioxide mitigation" OR "CO2 mitigation" OR "GHG mitigation" OR "greenhouse gas mitigation" OR "carbon sequestration" OR "CO2 sequestration" OR "carbon stock" OR "carbon storage" OR "carbon balance" OR "carbon accounting" OR "carbon emissions" OR "CO2 emissions" OR "carbon sink" OR "carbon fixation" OR "carbon density" OR "greenhouse gas emission" OR "GHG emission" OR "aboveground" OR "above-ground"AND(impact OR effect* OR evaluat* OR empiric* OR assess*)NOT | 8 |
| Environment Complete | 8/30/2021 | TI(forest OR woodland OR meadow OR pasture OR agricultur* OR rangeland OR grassland OR mangrove OR tree OR cropland OR grazing OR land OR ecosystem OR landscape OR rice OR tropic*) OR KW(forest OR woodland OR meadow OR pasture OR agricultur* OR rangeland OR grassland OR mangrove OR tree OR cropland OR grazing OR land OR ecosystem OR landscape OR rice OR tropic*) OR AB(forest OR woodland OR meadow OR pasture OR agricultur* OR rangeland OR grassland OR mangrove OR tree OR cropland OR grazing OR land OR ecosystem OR landscape OR rice OR tropic*)ANDTI(restoration OR reforestation OR afforestation OR replanting OR rehabilitation OR enrichment OR "tree islands" OR "rice production" OR "rice intensification" OR "rice cultivation" OR "community forest" OR "community forests" OR "community forestry" OR "shade grown" OR "climate-smart" OR "pasture management" OR "cover crop" OR "cover crops" OR "nutrient management" OR agroforestry OR agroforest OR silvopastor* OR silvopastur* OR silvo-pastor* OR silvo-pastur* OR agro-ecolog* OR agroecolog* OR "conservation agriculture" OR "tree planting" OR fencing OR exclosure OR ((partial OR selecti* OR gap OR retention) NEAR/3 (felling OR cutting OR harvest*)) OR "grazing management" OR "active management" OR "salvage logging" OR "reduced-impact logging" OR "alley cropping" OR "fire management" OR plantation OR "forest management" OR "manure management" OR ((crop OR cropland) NEAR/2 management) OR windbreaks OR thinning OR "protected area" OR "protected areas" OR ("Indigenous Peoples" OR "Indigenous communities" OR "Indigenous groups") OR "national park" OR "concession" OR "buffer zone" OR "sacred groves" OR "sacred forests" OR "sacred forest" OR "sacred grove" OR (protection NEAR/2 (forest OR landscape OR grassland)) OR "land stewardship" OR "natural climate solutions" OR "natural climate solution" OR "ecosystem-based adaptation" OR "carbon forestry" OR "payments for ecosystem services" OR "payments for environmental services" OR "PES" OR "REDD" OR "REDD+" OR "Reduced Emissions from Deforestation and Degradation" OR "sloping land conversion" OR "cropland to forest") OR KW(restoration OR reforestation OR afforestation OR replanting OR rehabilitation OR enrichment OR "tree islands" OR "rice production" OR "rice intensification" OR "rice cultivation" OR "community forest" OR "community forests" OR "community forestry" OR "shade grown" OR "climate-smart" OR "pasture management" OR "cover crop" OR "cover crops" OR "nutrient management" OR agroforestry OR agroforest OR silvopastor* OR silvopastur* OR silvo-pastor* OR silvo-pastur* OR agro-ecolog* OR agroecolog* OR "conservation agriculture" OR "tree planting" OR fencing OR exclosure OR ((partial OR selecti* OR gap OR retention) NEAR/3 (felling OR cutting OR harvest*)) OR "grazing management" OR "active management" OR "salvage logging" OR "reduced-impact logging" OR "alley cropping" OR "fire management" OR plantation OR "forest management" OR "manure management" OR ((crop OR cropland) NEAR/2 management) OR windbreaks OR thinning OR "protected area" OR "protected areas" OR ("Indigenous Peoples" OR "Indigenous communities" OR "Indigenous groups") OR "national park" OR "concession" OR "buffer zone" OR "sacred groves" OR "sacred forests" OR "sacred forest" OR "sacred grove" OR (protection NEAR/2 (forest OR landscape OR grassland)) OR "land stewardship" OR "natural climate solutions" OR "natural climate solution" OR "ecosystem-based adaptation" OR "carbon forestry" OR "payments for ecosystem services" OR "payments for environmental services" OR "PES" OR "REDD" OR "REDD+" OR "Reduced Emissions from Deforestation and Degradation" OR "sloping land conversion" OR "cropland to forest") OR AB(restoration OR reforestation OR afforestation OR replanting OR rehabilitation OR enrichment OR "tree islands" OR "rice production" OR "rice intensification" OR "rice cultivation" OR "community forest" OR "community forests" OR "community forestry" OR "shade grown" OR "climate-smart" OR "pasture management" OR "cover crop" OR "cover crops" OR "nutrient management" OR agroforestry OR agroforest OR silvopastor* OR silvopastur* OR silvo-pastor* OR silvo-pastur* OR agro-ecolog* OR agroecolog* OR "conservation agriculture" OR "tree planting" OR fencing OR exclosure OR ((partial OR selecti* OR gap OR retention) NEAR/3 (felling OR cutting OR harvest*)) OR "grazing management" OR "active management" OR "salvage logging" OR "reduced-impact logging" OR "alley cropping" OR "fire management" OR plantation OR "forest management" OR "manure management" OR ((crop OR cropland) NEAR/2 management) OR windbreaks OR thinning OR "protected area" OR "protected areas" OR ("Indigenous Peoples" OR "Indigenous communities" OR "Indigenous groups") OR "national park" OR "concession" OR "buffer zone" OR "sacred groves" OR "sacred forests" OR "sacred forest" OR "sacred grove" OR (protection NEAR/2 (forest OR landscape OR grassland)) OR "land stewardship" OR "natural climate solutions" OR "natural climate solution" OR "ecosystem-based adaptation" OR "carbon forestry" OR "payments for ecosystem services" OR "payments for environmental services" OR "PES" OR "REDD" OR "REDD+" OR "Reduced Emissions from Deforestation and Degradation" OR "sloping land conversion" OR "cropland to forest")ANDTI("land use change" OR "land-use change" OR "land conversion" OR "forest conversion" OR "grassland conversion" OR deforestation OR "land cover" OR "forest cover" OR "vegetation cover" OR "habitat cover" OR "tree cover" OR (clearing NEAR/4 (forest OR land)) OR ((diversity OR composition OR recovery OR succession) NEAR/1 (tree OR forest)) OR ((biomass OR biomasses) NEAR/2 (tree OR shrub OR woody OR aboveground OR above-ground OR recovery OR living)) OR (degradation NEAR/2 (forest OR grassland)) OR ((climate OR carbon OR CO2 OR GHG OR "greenhouse gas") NEAR/3 mitigat*) OR ((carbon OR CO2) NEAR/2 (sequestration OR balance OR accounting OR storage OR emission OR sink OR stock OR fixation OR density)) OR (("greenhouse gas" OR GHG) NEAR/2 (emission OR avoid* OR reduc*)) OR aboveground OR above-ground) OR KW("land use change" OR "land-use change" OR "land conversion" OR "forest conversion" OR "grassland conversion" OR deforestation OR "land cover" OR "forest cover" OR "vegetation cover" OR "habitat cover" OR "tree cover" OR (clearing NEAR/4 (forest OR land)) OR ((diversity OR composition OR recovery OR succession) NEAR/1 (tree OR forest)) OR ((biomass OR biomasses) NEAR/2 (tree OR shrub OR woody OR aboveground OR above-ground OR recovery OR living)) OR (degradation NEAR/2 (forest OR grassland)) OR ((climate OR carbon OR CO2 OR GHG OR "greenhouse gas") NEAR/3 mitigat*) OR ((carbon OR CO2) NEAR/2 (sequestration OR balance OR accounting OR storage OR emission OR sink OR stock OR fixation OR density)) OR (("greenhouse gas" OR GHG) NEAR/2 (emission OR avoid* OR reduc*)) OR aboveground OR above-ground) OR AB("land use change" OR "land-use change" OR "land conversion" OR "forest conversion" OR "grassland conversion" OR deforestation OR "land cover" OR "forest cover" OR "vegetation cover" OR "habitat cover" OR "tree cover" OR (clearing NEAR/4 (forest OR land)) OR ((diversity OR composition OR recovery OR succession) NEAR/1 (tree OR forest)) OR ((biomass OR biomasses) NEAR/2 (tree OR shrub OR woody OR aboveground OR above-ground OR recovery OR living)) OR (degradation NEAR/2 (forest OR grassland)) OR ((climate OR carbon OR CO2 OR GHG OR "greenhouse gas") NEAR/3 mitigat*) OR ((carbon OR CO2) NEAR/2 (sequestration OR balance OR accounting OR storage OR emission OR sink OR stock OR fixation OR density)) OR (("greenhouse gas" OR GHG) NEAR/2 (emission OR avoid* OR reduc*)) OR aboveground OR above-ground)ANDTI(impact OR effect* OR evaluat* OR empiric* OR assess*) OR KW(impact OR effect* OR evaluat* OR empiric* OR assess*) OR AB(impact OR effect* OR evaluat* OR empiric* OR assess*)NOTTI("Canada" OR "British Colombia" OR "Europe" OR "Sweden" OR "Norway" OR "Finland" OR Scandinavia* OR Mediterranean OR Chile OR "United Kingdom" OR Korea OR Pakistan OR Russia OR Denmark OR England OR Wales OR Ireland OR Scotland OR "integrated water resource management" OR European OR Spain OR Spanish OR Alabama OR Alaska OR Arizona OR Arkansas OR California OR Colorado OR Connecticut OR Delaware OR Florida OR Georgia OR Idaho OR Illinois OR Indiana OR Iowa OR Kansas OR Kentucky OR Louisiana OR Maine OR Maryland OR Massachusetts OR Michigan OR Minnesota OR Mississippi OR Missouri OR Montana OR Nebraska OR Nevada OR "New Hampshire" OR "New Jersey" OR "New Mexico" OR "New York" OR "North Carolina" OR "North Dakota" OR Ohio OR Oklahoma OR Oregon OR Pennsylvania OR "Rhode Island" OR "South Carolina" OR "South Dakota" OR Tennessee OR Texas OR Utah OR Vermont OR Virginia OR Washington OR "West Virginia" OR Wisconsin OR Wyoming OR peatland OR "north america" OR "north american" OR Albania OR Andorra OR Armenia OR Austria OR Azerbaijan OR Belarus OR Belgium OR Bosnia and Herzegovina OR Bulgaria OR Croatia OR Cyprus OR Czechia OR Estonia OR France OR Germany OR Greece OR Hungary OR Iceland OR Italy OR Kazakhstan OR Kosovo OR Latvia OR Liechtenstein OR Lithuania OR Luxembourg OR Malta OR Moldova OR Monaco OR Montenegro OR Netherlands OR Poland OR Portugal OR Romania OR Russia OR San Marino OR Serbia OR Slovakia OR Slovenia OR Switzerland OR Turkey OR Ukraine OR "Vatican City" OR Alberta OR "British Columbia" OR Manitoba OR "New Brunswick" OR Newfoundland OR "Northwest Territories" OR "Nova Scotia" OR Nunavut OR Ontario OR "Prince Edward Island" OR Quebec OR Saskatchewan OR Yukon OR Labrador) OR KW("Canada" OR "British Colombia" OR "Europe" OR "Sweden" OR "Norway" OR "Finland" OR Scandinavia* OR Mediterranean OR Chile OR "United Kingdom" OR Korea OR Pakistan OR Russia OR Denmark OR England OR Wales OR Ireland OR Scotland OR "integrated water resource management" OR European OR Spain OR Spanish OR Alabama OR Alaska OR Arizona OR Arkansas OR California OR Colorado OR Connecticut OR Delaware OR Florida OR Georgia OR Idaho OR Illinois OR Indiana OR Iowa OR Kansas OR Kentucky OR Louisiana OR Maine OR Maryland OR Massachusetts OR Michigan OR Minnesota OR Mississippi OR Missouri OR Montana OR Nebraska OR Nevada OR "New Hampshire" OR "New Jersey" OR "New Mexico" OR "New York" OR "North Carolina" OR "North Dakota" OR Ohio OR Oklahoma OR Oregon OR Pennsylvania OR "Rhode Island" OR "South Carolina" OR "South Dakota" OR Tennessee OR Texas OR Utah OR Vermont OR Virginia OR Washington OR "West Virginia" OR Wisconsin OR Wyoming OR peatland OR "north america" OR "north american" OR Albania OR Andorra OR Armenia OR Austria OR Azerbaijan OR Belarus OR Belgium OR Bosnia and Herzegovina OR Bulgaria OR Croatia OR Cyprus OR Czechia OR Estonia OR France OR Germany OR Greece OR Hungary OR Iceland OR Italy OR Kazakhstan OR Kosovo OR Latvia OR Liechtenstein OR Lithuania OR Luxembourg OR Malta OR Moldova OR Monaco OR Montenegro OR Netherlands OR Poland OR Portugal OR Romania OR Russia OR San Marino OR Serbia OR Slovakia OR Slovenia OR Switzerland OR Turkey OR Ukraine OR "Vatican City" OR Alberta OR "British Columbia" OR Manitoba OR "New Brunswick" OR Newfoundland OR "Northwest Territories" OR "Nova Scotia" OR Nunavut OR Ontario OR "Prince Edward Island" OR Quebec OR Saskatchewan OR Yukon OR Labrador) OR AB("Canada" OR "British Colombia" OR "Europe" OR "Sweden" OR "Norway" OR "Finland" OR Scandinavia* OR Mediterranean OR Chile OR "United Kingdom" OR Korea OR Pakistan OR Russia OR Denmark OR England OR Wales OR Ireland OR Scotland OR "integrated water resource management" OR European OR Spain OR Spanish OR Alabama OR Alaska OR Arizona OR Arkansas OR California OR Colorado OR Connecticut OR Delaware OR Florida OR Georgia OR Idaho OR Illinois OR Indiana OR Iowa OR Kansas OR Kentucky OR Louisiana OR Maine OR Maryland OR Massachusetts OR Michigan OR Minnesota OR Mississippi OR Missouri OR Montana OR Nebraska OR Nevada OR "New Hampshire" OR "New Jersey" OR "New Mexico" OR "New York" OR "North Carolina" OR "North Dakota" OR Ohio OR Oklahoma OR Oregon OR Pennsylvania OR "Rhode Island" OR "South Carolina" OR "South Dakota" OR Tennessee OR Texas OR Utah OR Vermont OR Virginia OR Washington OR "West Virginia" OR Wisconsin OR Wyoming OR peatland OR "north america" OR "north american" OR Albania OR Andorra OR Armenia OR Austria OR Azerbaijan OR Belarus OR Belgium OR Bosnia and Herzegovina OR Bulgaria OR Croatia OR Cyprus OR Czechia OR Estonia OR France OR Germany OR Greece OR Hungary OR Iceland OR Italy OR Kazakhstan OR Kosovo OR Latvia OR Liechtenstein OR Lithuania OR Luxembourg OR Malta OR Moldova OR Monaco OR Montenegro OR Netherlands OR Poland OR Portugal OR Romania OR Russia OR San Marino OR Serbia OR Slovakia OR Slovenia OR Switzerland OR Turkey OR Ukraine OR "Vatican City" OR Alberta OR "British Columbia" OR Manitoba OR "New Brunswick" OR Newfoundland OR "Northwest Territories" OR "Nova Scotia" OR Nunavut OR Ontario OR "Prince Edward Island" OR Quebec OR Saskatchewan OR Yukon OR Labrador) Limit source types to Academic Journals and language to English | 6706 |
| Web of Science (Core Collection) | 8/27/2021 | TS=(forest OR woodland OR meadow OR pasture OR agricultur* OR rangeland OR grassland OR mangrove OR tree OR cropland OR grazing OR land OR ecosystem OR landscape OR rice OR tropic*)AND(TS=(restoration OR reforestation OR afforestation OR replanting OR rehabilitation OR enrichment OR "tree islands") OR TS=("rice production" OR "rice intensification" OR "rice cultivation" OR "community forest" OR "community forests" OR "community forestry" OR "shade grown" OR "climate-smart" OR "pasture management" OR "cover crop" OR "cover crops" OR "nutrient management" OR agroforestry OR agroforest OR silvopastor* OR silvopastur* OR silvo-pastor* OR silvo-pastur* OR agro-ecolog* OR agroecolog* OR "conservation agriculture" OR "tree planting" OR fencing OR exclosure OR ((partial OR selecti* OR gap OR retention) NEAR/3 (felling OR cutting OR harvest*)) OR "grazing management" OR "active management" OR "salvage logging" OR "reduced-impact logging" OR "alley cropping" OR "fire management" OR plantation OR "forest management" OR "manure management" OR ((crop OR cropland) NEAR/2 management) OR windbreaks OR thinning) OR TS=("protected area" OR "protected areas" OR ("Indigenous Peoples" OR "Indigenous communities" OR "Indigenous groups") OR "national park" OR "concession" OR "buffer zone" OR "sacred groves" OR "sacred forests" OR "sacred forest" OR "sacred grove" OR (protection NEAR/2 (forest OR landscape OR grassland))) OR TS=("land stewardship" OR "natural climate solutions" OR "natural climate solution" OR "ecosystem-based adaptation" OR "carbon forestry" OR "payments for ecosystem services" OR "payments for environmental services" OR "PES" OR "REDD" OR "REDD+" OR "Reduced Emissions from Deforestation and Degradation" OR "sloping land conversion" OR "cropland to forest"))ANDTS=("land use change" OR "land-use change" OR "land conversion" OR "forest conversion" OR "grassland conversion" OR deforestation OR "land cover" OR "forest cover" OR "vegetation cover" OR "habitat cover" OR "tree cover" OR (clearing NEAR/4 (forest OR land)) OR ((diversity OR composition OR recovery OR succession) NEAR/1 (tree OR forest)) OR ((biomass OR biomasses) NEAR/2 (tree OR shrub OR woody OR aboveground OR above-ground OR recovery OR living)) OR (degradation NEAR/2 (forest OR grassland)) OR ((climate OR carbon OR CO2 OR GHG OR "greenhouse gas") NEAR/3 mitigat*) OR ((carbon OR CO2) NEAR/2 (sequestration OR balance OR accounting OR storage OR emission OR sink OR stock OR fixation OR density)) OR (("greenhouse gas" OR GHG) NEAR/2 (emission OR avoid* OR reduc*)) OR aboveground OR above-ground)ANDTS=(impact OR effect* OR evaluat* OR empiric* OR assess*)NOTTS=("Canada" OR "British Colombia" OR "Europe" OR "Sweden" OR "Norway" OR "Finland" OR Scandinavia* OR Mediterranean OR Chile OR "United Kingdom" OR Korea OR Pakistan OR Russia OR Denmark OR England OR Wales OR Ireland OR Scotland OR "integrated water resource management" OR European OR Spain OR Spanish OR Alabama OR Alaska OR Arizona OR Arkansas OR California OR Colorado OR Connecticut OR Delaware OR Florida OR Georgia OR Idaho OR Illinois OR Indiana OR Iowa OR Kansas OR Kentucky OR Louisiana OR Maine OR Maryland OR Massachusetts OR Michigan OR Minnesota OR Mississippi OR Missouri OR Montana OR Nebraska OR Nevada OR "New Hampshire" OR "New Jersey" OR "New Mexico" OR "New York" OR "North Carolina" OR "North Dakota" OR Ohio OR Oklahoma OR Oregon OR Pennsylvania OR "Rhode Island" OR "South Carolina" OR "South Dakota" OR Tennessee OR Texas OR Utah OR Vermont OR Virginia OR Washington OR "West Virginia" OR Wisconsin OR Wyoming OR peatland OR "north america" OR "north american" OR Albania OR Andorra OR Armenia OR Austria OR Azerbaijan OR Belarus OR Belgium OR Bosnia and Herzegovina OR Bulgaria OR Croatia OR Cyprus OR Czechia OR Estonia OR France OR Germany OR Greece OR Hungary OR Iceland OR Italy OR Kazakhstan OR Kosovo OR Latvia OR Liechtenstein OR Lithuania OR Luxembourg OR Malta OR Moldova OR Monaco OR Montenegro OR Netherlands OR Poland OR Portugal OR Romania OR Russia OR San Marino OR Serbia OR Slovakia OR Slovenia OR Switzerland OR Turkey OR Ukraine OR "Vatican City" OR Alberta OR "British Columbia" OR Manitoba OR "New Brunswick" OR Newfoundland OR "Northwest Territories" OR "Nova Scotia" OR Nunavut OR Ontario OR "Prince Edward Island" OR Quebec OR Saskatchewan OR Yukon OR Labrador) NOTTI=("modelling" OR "modeling") Limit source types to Articles, Reviews, Book chapters, Proceedings. English Language only. | 24,477 |
