# Supplementary material for: Assessing evidence on the impacts of nature-based interventions for climate change mitigation: a systematic map of primary and secondary research from subtropical and tropical terrestrial regions
Source: Environ Evid. 2023 Oct 25;12:21. doi: 10.1186/s13750-023-00312-3 (PMC11378798; doi:10.1186/s13750-023-00312-3)
Supplement: Supplementary file 11 — Additional file 11. ROSES Reporting Forms for systematic map. [file 13750_2023_312_MOESM11_ESM.docx]

Additional File 11 – ROSES Reporting Forms for systematic map

| **ROSES FORM for Systematic map** | |  |  |  |  |
| --- | --- | --- | --- | --- | --- |
| **Item number** | **Section/sub-section** | **Topic** | **Checklist/meta-data** | **Author response** | **Comments** |
| 1 | Title | Title | Meta-data | What evidence exists on the impacts of nature-based interventions for climate change mitigation? A systematic map of empirical evidence from subtropical and tropical terrestrial regions |  |
| 2 | Type of review | Type of review | Meta-data | systematic map |  |
| 3 | Authors' contacts | Authors' contacts | Checklist | Yes |  |
| 4 | Abstract | Structured summary | Checklist | Yes | 430 words |
| 5 | Background | Background | Checklist | Yes | Please see background and framework development section |
| 6 | Stakeholder engagement | Stakeholder engagement | Checklist | Yes | Please see methods section. |
| 7 | Objective of the review | Objective | Checklist | Yes | Please see objectives section. |
| 8 |  | Definition of the question components | Meta-data | PopulationWe focused on ecoregions within the following tropical and subtropical terrestrial biomes: Tropical and Subtropical Coniferous Forests; Tropical and Subtropical Dry Broadleaf Forests; Tropical and Subtropical Grasslands, Savannas & Shrublands; Tropical and Subtropical Moist Broadleaf Forests; Mangroves (Olson et al. 2001).Primary InterventionWe included interventions across three broad themes—protection, management, and restoration. We distinguish between forest and other land use management and agricultural management. See Additional File 4 for full typology of included interventions.Study type(s) We included primary research articles in English that met the following criteria:Non-experimental, quasi-experimental, and experimental study designs that use quantitative, qualitative, or a combination of data typesOutcomesWe included articles that assess changes in greenhouse gas (GHG) emissions, amount of carbon stored (carbon storage), and/or rates of carbon sequestration within aboveground biomass as an indicator of climate change mitigation. We also include articles that assess changes to land cover, land use type, and/or land condition as proxy indicators as they represent intermediate outcomes on the pathway towards mitigation. See Additional File 5 for a full typology of included outcomes. |  |
| 9 | Methods | Protocol | Meta-data | Cheng, Samantha H., et al. "What evidence exists on the links between natural climate solutions and climate change mitigation outcomes in subtropical and tropical terrestrial regions? A systematic map protocol." Environmental evidence 11.1 (2022): 1-17. https://doi.org/10.1186/s13750-022-00268-w |  |
| 10 |  | Deviations from protocol | Checklist | Yes | See methods section for list of any deviations from protocol. |
| 11 | Searches | Search strategy | Checklist | Yes | See methods as well as Additional File 2 Comprehensive Search Strategy Methods |
| 12 |  | Search string | Meta-data | TS=(forest OR woodland OR meadow OR pasture OR agricultur* OR rangeland OR grassland OR mangrove OR tree OR cropland OR grazing OR land OR ecosystem OR landscape OR rice OR tropic*) AND (TS=(restoration OR reforestation OR afforestation OR replanting OR rehabilitation OR enrichment OR "tree islands") OR TS=("rice production" OR "rice intensification" OR "rice cultivation" OR "community forest" OR "community forests" OR "community forestry" OR "shade grown" OR "climate-smart" OR "pasture management" OR "cover crop" OR "cover crops" OR "nutrient management" OR agroforestry OR agroforest OR silvopastor* OR silvopastur* OR silvo-pastor* OR silvo-pastur* OR agro-ecolog* OR agroecolog* OR "conservation agriculture" OR "tree planting" OR fencing OR exclosure OR ((partial OR selecti* OR gap OR retention) NEAR/3 (felling OR cutting OR harvest*)) OR "grazing management" OR "active management" OR "salvage logging" OR "reduced-impact logging" OR "alley cropping" OR "fire management" OR plantation OR "forest management" OR "manure management" OR ((crop OR cropland) NEAR/2 management) OR windbreaks OR thinning) OR TS=("protected area" OR "protected areas" OR ("Indigenous Peoples" OR "Indigenous communities" OR "Indigenous groups") OR "national park" OR "concession" OR "buffer zone" OR "sacred groves" OR "sacred forests" OR "sacred forest" OR "sacred grove" OR (protection NEAR/2 (forest OR landscape OR grassland))) OR TS=("land stewardship" OR "natural climate solutions" OR "natural climate solution" OR "ecosystem-based adaptation" OR "carbon forestry" OR "payments for ecosystem services" OR "payments for environmental services" OR "PES" OR "REDD" OR "REDD+" OR "Reduced Emissions from Deforestation and Degradation" OR "sloping land conversion" OR "cropland to forest")) AND TS=("land use change" OR "land-use change" OR "land conversion" OR "forest conversion" OR "grassland conversion" OR deforestation OR "land cover" OR "forest cover" OR "vegetation cover" OR "habitat cover" OR "tree cover" OR (clearing NEAR/4 (forest OR land)) OR ((diversity OR composition OR recovery OR succession) NEAR/1 (tree OR forest)) OR ((biomass OR biomasses) NEAR/2 (tree OR shrub OR woody OR aboveground OR above-ground OR recovery OR living)) OR (degradation NEAR/2 (forest OR grassland)) OR ((climate OR carbon OR CO2 OR GHG OR "greenhouse gas") NEAR/3 mitigat*) OR ((carbon OR CO2) NEAR/2 (sequestration OR balance OR accounting OR storage OR emission OR sink OR stock OR fixation OR density)) OR (("greenhouse gas" OR GHG) NEAR/2 (emission OR avoid* OR reduc*)) OR aboveground OR above-ground) AND TS=(impact OR effect* OR evaluat* OR empiric* OR assess*) NOT TS=("United States" OR "Canada" OR "British Colombia" OR "Europe" OR "Sweden" OR "Norway" OR "Finland" OR Scandinavia* OR urban OR city OR cities OR Mediterranean OR Japan OR Argentina OR Chile OR "South Africa" OR "United Kingdom" OR Korea OR Pakistan OR Russia OR Denmark OR England OR Wales OR Ireland OR Scotland OR "integrated water resource management" OR European OR Spain OR Spanish OR USA OR Alabama OR Alaska OR Arizona OR Arkansas OR California OR Colorado OR Connecticut OR Delaware OR Florida OR Georgia OR Idaho OR Illinois OR Indiana OR Iowa OR Kansas OR Kentucky OR Louisiana OR Maine OR Maryland OR Massachusetts OR Michigan OR Minnesota OR Mississppi OR Missouri OR Montana OR Nebraska OR Nevada OR "New Hampshire" OR "New Jersey" OR "New Mexico" OR "New York" OR "North Carolina" OR "North Dakota" OR Ohio OR Oklahoma OR Oregon OR Pennsylvania OR "Rhode Island" OR "South Carolina" OR "South Dakota" OR Tennessee OR Texas OR Utah OR Vermont OR Virginia OR Washington OR "West Virginia" OR Wisconsin OR Wyoming OR peatland OR "north america" OR "north american" OR Albania OR Andorra OR Armenia OR Austria OR Azerbaijan OR Belarus OR Belgium OR Bosnia and Herzegovina OR Bulgaria OR Croatia OR Cyprus OR Czechia OR Estonia OR France OR Germany OR Greece OR Hungary OR Iceland OR Italy OR Kazakhstan OR Kosovo OR Latvia OR Liechtenstein OR Lithuania OR Luxembourg OR Malta OR Moldova OR Monaco OR Montenegro OR Netherlands OR Poland OR Portugal OR Romania OR Russia OR San Marino OR Serbia OR Slovakia OR Slovenia OR Switzerland OR Turkey OR Ukraine OR "Vatican City" OR Alberta OR "British Columbia" OR Manitoba OR "New Brunswick" OR Newfoundland OR "Northwest Territories" OR "Nova Scotia" OR Nunavut OR Ontario OR "Prince Edward Island" OR Quebec OR Saskatchewan OR Yukon OR Labrador OR "U.S." OR "U.S.A." OR "U.K." OR "UK" OR "USA") NOT TI=("modelling" OR "modeling") | Web of Science |
| 13 |  | Languages - bibliographic databases | Meta-data | English |  |
| 14 |  | Languages – grey literature | Meta-data | English |  |
| 15 |  | Bibliographic databases | Meta-data | 3 |  |
| 16 |  | Web-based search engines | Meta-data | 0 |  |
| 17 |  | Organisational websites | Meta-data | 68 |  |
| 18 |  | Estimating comprehensiveness of the search | Checklist | Yes | Yes, see Additional file 2 of protocol for test library (Cheng et al. 2022) |
| 19 |  | Search update | Checklist | No | N/A - Searches were performed less than 2 years ago (August 2021) |
| 20 | Article screening and study inclusion criteria | Screening strategy | Checklist | Yes | See "Article screening and study eligibility criteria in the Methods |
| 21 |  | Inclusion criteria | Checklist | Yes | See "Article screening and study eligibility criteria in the Methods |
| 22 | Critical appraisal | Critical appraisal strategy | Checklist | No | Given the large size of this systematic map we did not undertake a quality assessment for individual articles in terms of reliability and relevance based on study design. |
| 23 |  | Critical appraisal used in synthesis | Checklist | No |  |
| 24 | Meta-data extraction and coding strategy | Meta-data extraction and coding strategy | Checklist | Yes | See "Data coding strategy" in Methods section |
| 25 |  | Approaches to missing data | Checklist | No | N/A |
| 26 | Data synthesis and presentation | Narrative synthesis strategy | Checklist | Yes | See "Data coding strategy" in Methods section |
| 27 |  | Knowledge gap and cluster identification strategy | Checklist | Yes | See "Data mapping method" in Methods section |
| 28 |  | Demonstrating procedural independence | Checklist | Yes | See "Additional File 2 - Comprehensive Search Strategy Methods" |
| 29 | Results (review findings) | Description of review process | Checklist | Yes | See "Number and Type of Articles" in Results section |
| 30 |  | Number of search results | Meta-data | 35,435 |  |
| 31 |  | Number of search results after duplicate removal | Meta-data | 29,235 |  |
| 32 |  | Full text screening excludes | Checklist | Yes | See Additional File 8 - Excluded Studies |
| 33 |  | Title screening results | Meta-data | N/A |  |
| 34 |  | Abstract screening results | Meta-data | N/A |  |
| 35 |  | Title and abstract screening results | Meta-data | 2,294 |  |
| 36 |  | Retrieval results | Meta-data | 2,189 |  |
| 37 |  | Unobtainable articles | Checklist | Yes | See Additional "File 8 - Excluded Studies" for papers with the following exclusion reasons: "No access to PDF" and "Could not locate PDF" |
| 38 |  | Full text screening results | Meta-data | 948 |  |
| 39 |  | Consistency checking: screening | Checklist | Yes | See "Screening process" in Methods section |
| 40 |  | Narrative synthesis | Checklist | Yes | See "Characteristics of the evidence base" in the Methods section |
| 41 |  | Systematic map database | Checklist | Yes | See Additional File 6 |
| 42 |  | Limitations of the review | Checklist | Yes | See "Limitations of the map" in the Discussion section |
| 43 |  | Limitations of the evidence base | Checklist | Yes | See "Limitations of the map" in the Discussion section |
| 44 | Conclusions | Knowledge gaps and clusters | Checklist | Yes | See Discussion section |
| 45 |  | Implications for policy/management | Checklist | Yes | See "Implications for Policy/Management" in Conclusion section |
| 46 |  | Implications for research | Checklist | Yes | See "Implications for Research" in Conclusion section |
| 47 | Declarations | Competing interests | Checklist | Yes | See "Competing Interests" section |
