# Supplementary material for: Assessing evidence on the impacts of nature-based interventions for climate change mitigation: a systematic map of primary and secondary research from subtropical and tropical terrestrial regions
Source: Environ Evid. 2023 Oct 25;12:21. doi: 10.1186/s13750-023-00312-3 (PMC11378798; doi:10.1186/s13750-023-00312-3)
Supplement: Supplementary file 9 — Additional file 9: Supplementary Figures and Tables Figure S1. ROSES flow diagram for map of reviews. Figure S2. Growth in peer-reviewed publications, grey literature, and reviews since 1990. Figure S3. Affiliation type of first authors of included articles. Figure S4. Types of comparators employed in peer-reviewed articles and grey literature across different types of outcomes measured and disaggregated by study design. Figure S5. Geographic scale of study of articles within systematic map and map of reviews. Figure S6. Distribution and occurrence of review articles examining links between nature-based interventions and co-impacts. Reviews included did not have to focus on links to climate change mitigation outcomes. Figure S7. Distribution and frequency of complementary actions implemented alongside nature-based interventions to address enabling conditions. Articles can appear in more than one complementary action type. Figure S8. Number of articles in the evidence map reporting cost information disaggregated by publication year. Figure S9: Distribution and frequency of articles measuring changes to co-benefits assessed within nature-based interventions. Articles can appear in more than one co-benefit type. Table S1. Distribution of categories of nature-based interventions examined across biome types in the systematic map. Articles can examine more than one pathway type and more than one biome. Table S2. Distribution of categories of nature-based interventions examined by geographic region in the systematic map. Articles can examine more than one pathway type and more than one region. Table S3. Distribution of included articles across intervention and outcome linkages, disaggregated by study design type (complementary to Fig. 15). [file 13750_2023_312_MOESM9_ESM.docx]

Additional File 9 - Figures and Tables

This file includes the supplemental figures and tables referenced in the main report.

**Figure S1.** ROSES flow diagram for map of reviews

**Figure S2.** Growth in peer-reviewed publications, grey literature, and reviews since 1990

**Figure S3.** Affiliation type of first authors of included articles

**Figure S4.** Types of comparators employed in peer-reviewed articles and grey literature across different types of outcomes measured and disaggregated by study design.

**Figure S5.** Geographic scale of study of articles within systematic map and map of reviews

**Figure S6.** Distribution and occurrence of review articles examining links between nature-based interventions and co-impacts. Reviews included did not have to focus on links to climate change mitigation outcomes.

**Figure S7.** Distribution and frequency of complementary actions implemented alongside nature-based interventions to address enabling conditions. Articles can appear in more than one complementary action type.

**Figure S8.** Number of articles in the evidence map reporting cost information disaggregated by publication year.

**Figure S9**: Distribution and frequency of articles measuring changes to co-benefits assessed within nature-based interventions. Articles can appear in more than one co-benefit type.

**Table S1.** Distribution of categories of nature-based interventions examined across biome types in the systematic map. Articles can examine more than one pathway type and more than one biome

|  | **Agricultural Management** | **Forest and Other Land Use Management** | **Protection** | **Restoration** | *Total articles* |
| --- | --- | --- | --- | --- | --- |
| **Mangroves** | 3 | 15 | 16 | 28 | *53* |
| **Coniferous** | 14 | 37 | 20 | 5 | *66* |
| **Dry Broadleaf** | 22 | 59 | 56 | 17 | *134* |
| **Grasslands** | 52 | 86 | 67 | 45 | *211* |
| **Moist broadleaf** | 173 | 276 | 187 | 177 | *679* |

**Table S2.** Distribution of categories of nature-based interventions examined by geographic region in the systematic map. Articles can examine more than one pathway type and more than one region

|  | **Agricultural Management** | **Forest and Other Land Use Management** | **Protection** | **Restoration** | *Total articles* |
| --- | --- | --- | --- | --- | --- |
| **Africa** | 68 | 96 | 60 | 38 | *217* |
| **Asia** | 85 | 152 | 95 | 97 | *370* |
| **Latin America** | 86 | 129 | 111 | 114 | *381* |
| **Oceania** | 2 | 8 | n/a | 10 | *20* |

**Note the one US study took place in Hawaii, which is a part of Oceania as the table reflects*

**Table S3.** Distribution of included articles across intervention and outcome linkages, disaggregated by study design type (complementary to Figure 15).

|  | **Climate change mitigation outcome** | | | |
| --- | --- | --- | --- | --- |
| **NbI type** | Carbon storage and sequestration | Greenhouse gas emissions | Land condition | Land use/Land cover |
| Conservation agriculture | Experiment = 4  Quasi-experimental = 7  Non-experimental = 11 | Experiment = 11  Quasi-experimental = 2  Non-experimental = 1 | Experiment = 5  Quasi-experimental = 7  Non-experimental = 8 | Experiment = 2  Quasi-experimental = 7  Non-experimental = 9 |
| Creating new ecosystems | Experiment = 7  Quasi-experimental = 8  Non-experimental = 12 | Experiment = 1  Quasi-experimental = 0  Non-experimental = 1 | Experiment = 5  Quasi-experimental = 10  Non-experimental = 9 | Experiment = 0  Quasi-experimental = 3  Non-experimental = 7 |
| Grazing - optimal intensity | Experiment = 2  Quasi-experimental = 1  Non-experimental = 0 | Experiment = 0  Quasi-experimental = 1  Non-experimental = 0 | Experiment = 4  Quasi-experimental = 4  Non-experimental = 1 | Experiment = 0  Quasi-experimental = 3  Non-experimental = 0 |
| Improved rice cultivation |  | Experiment = 6  Quasi-experimental = 1  Non-experimental = 1 |  |  |
| Manure management | Experiment = 1  Quasi-experimental = 0  Non-experimental = 0 | Experiment = 1  Quasi-experimental = 0  Non-experimental = 0 | Experiment = 1  Quasi-experimental = 0  Non-experimental = 0 |  |
| Natural resource management | Experiment = 17  Quasi-experimental = 39  Non-experimental = 25 | Experiment = 2  Quasi-experimental = 8  Non-experimental = 8 | Experiment = 24  Quasi-experimental = 55  Non-experimental = 38 | Experiment = 5  Quasi-experimental = 65  Non-experimental = 37 |
| Nutrient management | Experiment = 8  Quasi-experimental = 5  Non-experimental = 8 | Experiment = 7  Quasi-experimental = 4  Non-experimental = 3 | Experiment = 5  Quasi-experimental = 3  Non-experimental = 1 | Experiment = 1  Quasi-experimental = 0  Non-experimental = 1 |
| Protection | Experiment = 3  Quasi-experimental = 11  Non-experimental = 16 | Experiment = 0  Quasi-experimental = 2  Non-experimental = 5 | Experiment = 3  Quasi-experimental = 36  Non-experimental = 35 | Experiment = 5  Quasi-experimental = 93  Non-experimental = 84 |
| Restoration of existing ecosystems | Experiment = 14  Quasi-experimental = 24  Non-experimental = 26 | Experiment = 0  Quasi-experimental = 1  Non-experimental = 5 | Experiment = 36  Quasi-experimental = 63  Non-experimental = 54 | Experiment = 5  Quasi-experimental = 13  Non-experimental = 28 |
| Site/area management | Experiment = 7  Quasi-experimental = 9  Non-experimental = 17 | Experiment = 0  Quasi-experimental = 0  Non-experimental = 3 | Experiment = 9  Quasi-experimental = 21  Non-experimental = 37 | Experiment = 7  Quasi-experimental = 15  Non-experimental = 51 |
| Trees in croplands | Experiment = 12  Quasi-experimental = 46  Non-experimental = 42 | Experiment = 0  Quasi-experimental = 1  Non-experimental = 6 | Experiment = 6  Quasi-experimental = 53  Non-experimental = 32 | Experiment = 1  Quasi-experimental = 15  Non-experimental = 7 |
